# Analysis of Drug Resistance Characteristics and Risk Factors of Cavitary Tuberculosis Based on Whole Genome Sequencing and Machine Learning

**DOI:** 10.1093/ofid/ofag328

**Published:** 2026-06-03

**Authors:** Qingpeng Yang, Zhouhua Xie, Jing Ye, Wenyi Dong, Yan Huang, Huifang Qin, Chongxing Zhou, Liwen Huang, Jin Ou, Yue Chang, Zhezhe Cui

**Affiliations:** School of Public Health, The key Laboratory of Environmental Pollution Monitoring and Disease Control, Ministry of Education, Guizhou Medical University, Guiyang, China; The Fourth People's Hospital of Nanning City, Nanning, China; Guangxi Key Discipline Platform of Tuberculosis Control, Guangxi Center for Disease Control and Prevention, Nanning, China; Department of Science and Education, The Fourth People's Hospital of Nanning City, Nanning, China; Guangxi Key Discipline Platform of Tuberculosis Control, Guangxi Center for Disease Control and Prevention, Nanning, China; Guangxi Key Discipline Platform of Tuberculosis Control, Guangxi Center for Disease Control and Prevention, Nanning, China; Guangxi Key Discipline Platform of Tuberculosis Control, Guangxi Center for Disease Control and Prevention, Nanning, China; Guangxi Key Discipline Platform of Tuberculosis Control, Guangxi Center for Disease Control and Prevention, Nanning, China; Guangxi Key Discipline Platform of Tuberculosis Control, Guangxi Center for Disease Control and Prevention, Nanning, China; School of Medicine and Health Management, Center of Medicine Economics and Management Research, Guizhou Medical University, Guiyang, China; School of Public Health, The key Laboratory of Environmental Pollution Monitoring and Disease Control, Ministry of Education, Guizhou Medical University, Guiyang, China; Guangxi Key Discipline Platform of Tuberculosis Control, Guangxi Center for Disease Control and Prevention, Nanning, China

**Keywords:** cavity, machine learning, *Mycobacterium tuberculosis*, risk factors, whole-genome sequencing

## Abstract

**Background:**

Pulmonary cavitation, a high-burden driver of pulmonary tuberculosis (PTB) transmission, necessitates targeted interventions. This study comprehensively analyzes the drug-resistance profiles and risk factors associated with cavitary TB to inform precise prevention and control strategies.

**Methods:**

This study analyzed 1247 cavitary PTB patients from Guangxi (2020–2024) with complete strain and clinical data. Whole-genome sequencing (WGS) characterized strain lineages and drug resistance. Key predictors were selected using Lasso regression, and the optimal model was identified from 9 machine learning (ML) models based on AUC, Shapley Additive Explanations (SHAP) elucidated feature contributions to severe cavitary TB risk.

**Results:**

Lineages were primarily classified as Lineage 2 (65.20%), Lineage 4 (29.91%), and 35 cases of mixed infections (2.81%). Concordance between WGS and phenotypic drug susceptibility testing was moderate for Isoniazid (INH) (*κ* = 0.634; *χ*^2^ = 32.667, *P* < .001) but good for rifampicin (RFP) (*κ* = 0.774; difference not significant). Predominant mutations were rpoB_p.Ser450Leu (RFP), katG_p.Ser315Thr (INH), embB_p.Met306Val (Ethambutol, EMB), and rpsL_p.Lys43Arg (Streptomycin, S). Lasso regression selected 10 variables: fatigue, fever, history of previous TB treatment, gender, age, RFP, INH, occupation, S, and ethnicity. After evaluating 9 ML models, the Gradient Boosting Machine was selected as optimal. SHAP analysis identified fatigue, older age, history of TB treatment, fever, male, and rpoB_p.Ser450 mutation were positively associated with severe cavity formation.

**Conclusions:**

The rpoB_p.Ser450 mutation is linked to severe cavitary PTB, but clinical and population studies are still needed to confirm this association. Therefore, new tools based on clinical indicators and biomarkers are needed to achieve early warning and timely intervention for the risk of severe cavitation.

## Teaser

This study of 1,247 cavitary tuberculosis patients in Guangxi (2020–2024) identifies the rpoB_p.Ser450 mutation, along with fatigue and older age, as factors associated with severe cavitary disease. The findings of this study overturns the traditional understanding that the rpoB p.Ser450 mutation merely leads to drug resistance, and suggests it may enhance the pulmonary invasiveness of *Mycobacterium tuberculosis*.

Tuberculosis (TB) is caused by infection with *Mycobacterium tuberculosis* (MTB) and primarily affects the lungs [1]. In the 2024 Global Tuberculosis Report released by WHO, TB may once again become the leading cause of death from a single infectious disease worldwide. Among the 30 countries with high TB burden, China ranks third in estimated TB incidence (6.8%) [2]. In 2023, there were 400 000 cases of global multidrug-resistant tuberculosis (MDR-TB)/rifampicin-resistant tuberculosis (RR-TB), but the treatment success rate was only 68.00% [2]. In comparison, China's successful treatment rate was 66%, slightly lower than the global average [2]. Compared with drug-susceptible tuberculosis, drug-resistant tuberculosis (DR-TB), particularly MDR-TB/RR-TB, is characterized by greater transmissibility, longer treatment duration, and higher therapeutic complexity [3]. Consequently, the prevention and control of DR-TB continue to pose a significant challenge.

Guangxi region, located in southwestern China, faces a severe TB epidemic due to its relatively underdeveloped economy, high population density, and limited healthcare resources. As a result, the region not only bears a substantial TB burden but also exhibits an incidence rate markedly higher than the national average [4, 5]. Pulmonary cavity is one of the most common clinical features of TB, accounting for 44% of adult TB cases at diagnosis [6]. In cavitary TB, necrotic and liquefied lung tissue is expelled through bronchi, forming cavities containing gas and necrotic material. MTB within these cavities is prone to developing drug resistance [7]. Moreover, the substantial survival of latent MTB within the cavities, which persistently releases bacteria into the environment, similarly prolongs the disease course in MDR-TB patients and increases the recurrence rate of treatment failure [8], this phenotype is typically associated with a high MTB burden and enhanced transmissibility [9, 10], and also elevates the risk of secondary infections [11] anti-TB treatment, the presence of cavities provides favorable conditions for the colonization and growth of MTB, leading to an increased likelihood of drug resistance [12]. It has been reported that TB patients with diabetes are more prone to developing multiple cavities and thick-walled cavities, with their immune dysfunction potentially contributing to the formation of pulmonary cavities [13, 14]. Research findings indicate that baseline CD3^+^ T-cell counts and related immune status may have a potential impact on cavity closure in MDR-TB/RR-TB patients [15]. Furthermore, delayed diagnosis and treatment in patients with pulmonary tuberculosis (PTB) increase the risk of lung cavity formation [16, 17]. Therefore, conducting in-depth research on this key population of cavitary PTB patients is of critical importance for interrupting transmission chains, reducing outbreaks, and improving patient prognosis.

WGS identifies all genetic variations associated with drug resistance and species/family identification by analyzing the complete bacterial genome in a single analysis [18, 19]. Therefore, this study performed WGS on cavitary PTB patients from Guangxi to characterize their strain lineages and drug-resistance profiles. By integrating epidemiological features and applying ML, we selected the optimal predictive model, and Shapley Additive Explanations (SHAP) was applied to the best model to elucidate key associated features. This approach identified critical determinants influencing cavity formation, providing valuable insights into the pathogenesis of pulmonary cavities and enabling the development of differentiated prevention and control strategies.

## METHOD

### Study Design and Sample Inclusion

This study adopted a retrospective observational research design. Stratified by geographical attributes, 5 cities were randomly selected from the east, west, south, north and center in Guangxi region of southern China as monitoring points. From 2020 to 2024, patients with PTB cavities were collected using a continuous inclusion method. The inclusion criteria were as follows: (1) confirmed diagnosis of PTB, defined by positive MTB detection through culture and/or molecular assays [20]; (2) confirmed presence of pulmonary cavities through imaging examination; (3) the corresponding sputum culture strains were well preserved and underwent whole-genome sequencing; (4) available questionnaire information was available. The exclusion criteria were as follows: (1) no pulmonary cavity lesions were detected by imaging examination; (2) the strains were contaminated or of poor quality; (3) the corresponding questionnaire content was lacking.

**Figure 1.**
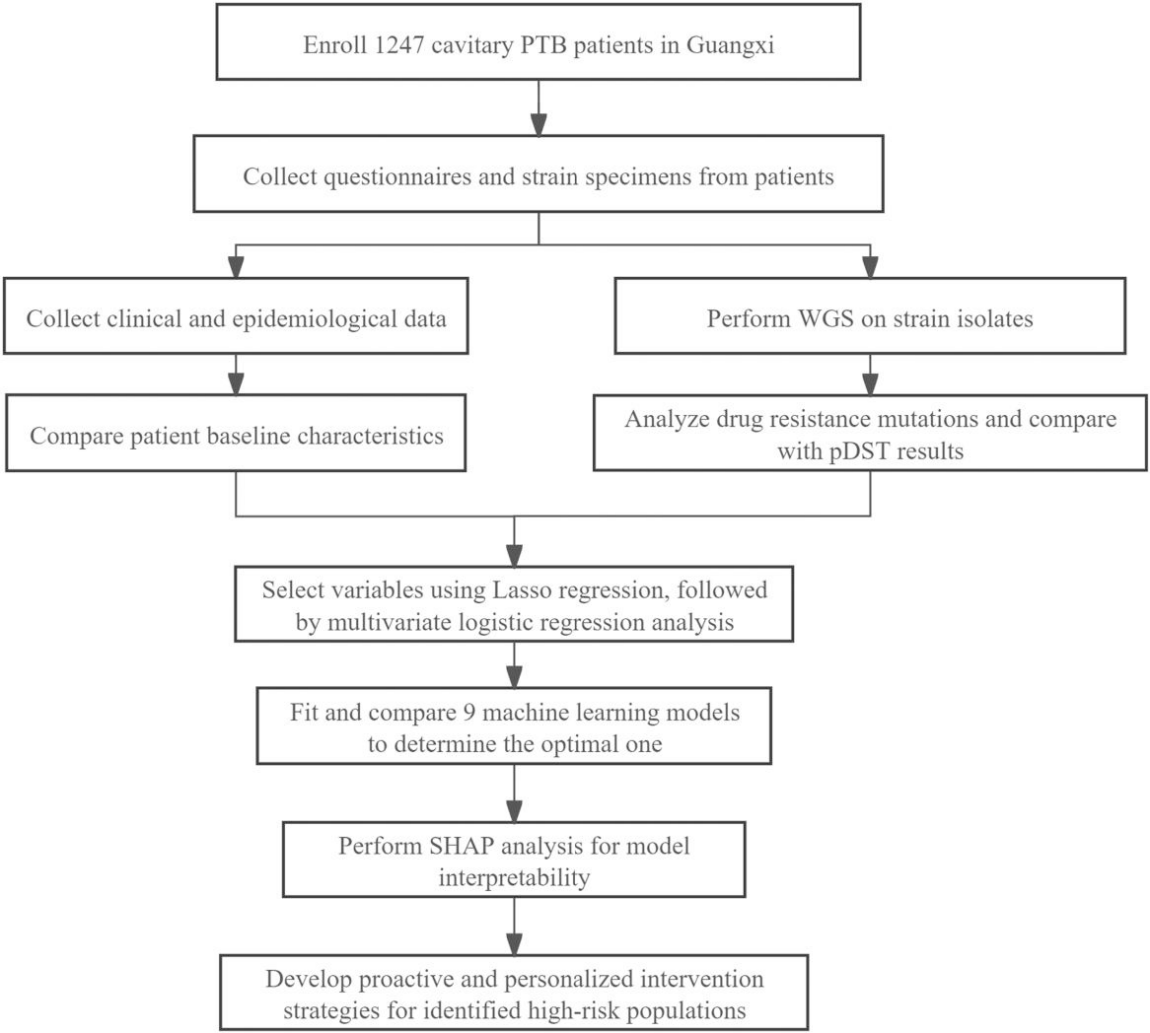
Flowchart of risk factor analysis study in patients with cavitary PTB.

The sample size was calculated using the infinite population proportion formula: *N* = $\frac{\;p(1-p)Z_{1-\frac{\alpha }{2}}^{2}}{d^{2}}$, *N*_Adjust_ = *N* * *deff*. In the pre-experiment, a total of 67 drug-resistant bacterial strains with mutations in the RRDR region were collected, among which 45 strains had mutations at the rpoB_p.Ser450 and rpoB_p.His445 sites, accounting for 67.16% (*p*). In the formula, Error (*d*) = 0.05, Alpha (α) = 0.05, *Z* = 1.96. Since this study adopted cluster sampling, *deff* = 2.00. The required sample size calculated by the formula was 680 cases (at least 136 cases per monitoring point). The actual number of patients included in the 5 monitoring points (east, south, west, north, and center) in this study was 358, 304, 186, 167, and 232 respectively, totaling 1247 cases. The flowchart is shown in Figure 1.

### Whole-Genome Sequencing

A total of 1247 clinical MTB isolates cultured on Löwenstein–Jensen medium were enrolled. DNA was extracted from each isolate and subjected to high-throughput Illumina next-generation sequencing. Paired-end 150 bp sequencing was performed with a minimum sequencing depth of 100×, and each sample generated no >1 Gb of clean data. Raw sequencing reads were filtered to remove adapter sequences and low-quality reads; the resulting clean data were used for subsequent bioinformatic analysis. Sequence analysis of MTB was performed using TB-Profiler v6.6.6. This software integrates sequence alignment, single nucleotide polymorphism (SNP) identification, genotyping, and drug-resistance prediction. It automatically detects SNP sites and determines the genotype without additional manual operations. Meanwhile, it matches detected variants against its built-in drug-resistance mutation database to accurately characterize mutation profiles associated with first-line antituberculosis drugs, thereby supporting source tracing and transmission chain analysis of infections.

### Traditional Phenotypic Drug Susceptibility Testing

The phenotypic drug susceptibility testing (pDST) for first-line antituberculosis drugs was performed using the conventional solid-medium-based method. The drug culture medium provided by Zhuhai BASO Biotechnology Co., Ltd. was employed for the assay. The concentrations of the tested drugs were set according to the *Technical Report on critical concentrations for drug susceptibility testing of medicines used in the treatment of drug-resistant tuberculosis* published by the World Health Organization [21]. Specifically, the concentrations were as follows: isoniazid (INH, 0.2 µg/mL), rifampicin (RFP, 40 µg/mL), ethambutol (EMB, 2 µg/mL), pyrazinamide (PZA, 100 µg/mL, pH 5.5), and streptomycin (S, 4 µg/mL). Strict quality control measures were implemented throughout the entire pDST process to ensure the reliability and accuracy of the experimental results, with detailed procedures provided in Supplementary material.

### Model Development and Comparison

Lasso regression was used to select the feature variables, and a multifactor logistic backward stepwise regression analysis was conducted on the feature variables. The data were divided into a training set and a test set in a 7:3 ratio. The class distribution was relatively balanced (∼1.5:1). A category weighting strategy was adopted for some models to reduce potential imbalance bias. The frequency of positive outcomes in the training set was 41.06% (358/872), and the frequency of positive outcomes in the test set was 37.60% (141/375). Nine ML models, including CatBoost, Random Forest (RF), EXtreme gradient boosting (XGboost), K-Nearest Neighbors (KNN), Gradient Boosting Machine (GBM), Neural Network (NN), Logistic, Support Vector Machine (SVM), and LightGBM, were fitted using the training data. Detailed variable assignments are provided in Supplementary Table 1. And the 5-fold cross-validation (CV) method and grid search method were used to find the optimal hyperparameters of the models. The Hosmer–Lemeshow test was used to evaluate the calibration of the model by analyzing the calibration curve, decision curve analysis (DCA) was carried out to evaluate the utility of the decision models. And The SHAP method offered global and local explanations for the model explanation.

### Statistical Analysis

Patients were divided into 2 groups based on the number of cavities according to chest imaging results: severe cavity group (≥3 cavities) and mild-to-moderate cavity group (<3 cavities) [22, 23]. Statistical analysis was performed using R 4.4.3 software. The count data were presented as “n or %”, and the measurement data were presented as “X ± S” according to the normal distribution, and comparisons were conducted using (*χ*^2^ test, Fisher's exact probability method, or Wilcoxon rank sum test). In addition, the odds ratio (OR) and 95% confidence interval (CI) were utilized to express the statistical results. The missing data situation for the variables is as follows: 15 cases (1.20%) of “Treatment” were missing, 8 cases (0.64%) of “Age” were missing, 125 cases (10.02%) of “Educational level” were missing, and 137 cases (10.99%) of “Chest Pain” were missing. The “Mice” package was used to perform multiple imputations for the missing data. All relevant covariates (including predictor factors, dependent variables, and other variables not included in the prediction model) were included in the imputation model for missing values. The imputation was conducted 5 times (following the Rubin rule). Binary variables were imputed using the logistic regression, ordinal multiclass variables were imputed using the proportional odds model, and continuous variables were imputed using the predictive mean matching method, the interpolation model is robust (as detailed in Supplementary Table 2). Image processing was performed using R 4.4.3 and the Chiplot website (access link: https://www.chiplot.online). Differences in the AUC values between 2 ROC curves were assessed using DeLong's test. A significance level of *α* = 0.05 was set, with two-sided *P* < .05 considered statistically significant.

### Ethical Review

This study has been approved by the Ethics Committee of the Guangxi Zhuang Autonomous Region Center for Disease Control and Prevention (GXRIB 2022-0011). The study was executed by the ethical standards set in the 1964 Declaration of Helsinki and its later modifications. The requirement for written informed consent was waived due to the retrospective design of this observational study.

## RESULTS

### Patient Characteristics

Among the 1247 enrolled patients, males predominated (82.60%, 1030/1247) with females accounting for 217 cases (17.40%). The mean age was 52.98 ± 16.47 years. Occupation was predominantly farmer (969/1247), while 29.11% (363/1247) were retreatment patients. Patients were classified into severe cavity group (≥3 cavities) and mild-to-moderate cavity group (<3 cavities) based on cavity count. Univariate analysis revealed statistically significant differences in age, treatment, gender, diabetes, fever, loss of appetite, fatigue, history of previous TB treatment, and RFP resistance mutations (*P* < .05). Furthermore, patients with the rpoB_p.Ser450 mutation had a higher proportion in the severe cavity group compared to the mild to mild-moderate cavity group (OR (95% CI): 1.86 (1.25–2.77), *P* = .002). No statistically significant differences were observed in lineage, INH resistance mutation type, EMB resistance mutation type, PZA resistance mutation type, S resistance mutation type, residential type, hepatitis, HIV, cough, chest pain, or night sweats (*P* > .05). Detailed results are presented in Table 1.

**Table 1.** Univariate Analysis of Clinical Characteristics in Cavitary PTB

Variable	Levels	Groups		OR (95% CI)	*P* Value
		Mild-to-moderate Cavity Group (*n* = 748)	Severe Cavity Group (*n* = 499)		
Age	-	51.01 ± 17.04	55.93 ± 15.12	1.02 (1.01–1.03)	<.001
Treatment	Initial treatment	565 (75.53)	319 (63.93)	1.00	-
	Retreatment	183 (24.47)	180 (36.07)	1.74 (1.36–2.23)	<.001
Residence	Town/City	173 (23.13)	116 (23.25)	1.00	-
	Rural area	575 (76.87)	383 (76.75)	0.99 (0.76–1.30)	.961
Gender	Male	601 (80.35)	429 (85.97)	1.00	-
	Female	147 (19.65)	70 (14.03)	0.67 (0.49–0.91)	.013
Diabetes	Not	603 (80.61)	371 (74.35)	1.00	-
	Yes	145 (19.39)	128 (25.65)	1.43 (1.09–1.88)	.009
Hepatitis	Not	721 (96.39)	483 (96.79)	1.00	-
	Yes	27 (3.61)	16 (3.21)	0.88 (0.47–1.66)	.702
HIV	Not	738 (98.66)	493 (98.80)	1.00	-
	Yes	10 (1.34)	6 (1.20)	0.90 (0.32–2.49)	.836
Cough	Not	64 (8.56)	42 (8.42)	1.00	-
	Yes	684 (91.44)	457 (91.58)	1.02 (0.68–1.53)	.931
Hemoptysis	Not	685 (91.58)	455 (91.18)	1.00	-
	Yes	63 (8.42)	44 (8.82)	1.05 (0.70–1.57)	.807
Fever	Not	635 (84.89)	389 (77.96)	1.00	-
	Yes	113 (15.11)	110 (22.04)	1.59 (1.19–2.13)	.002
Chest pain	Not	689 (92.11)	461 (92.38)	1.00	-
	Yes	59 (7.89)	38 (7.62)	0.96 (0.63–1.47)	.860
Night sweats	Not	689 (92.11)	461 (92.38)	1.00	-
	Yes	59 (7.89)	38 (7.62)	0.96 (0.63–1.47)	.860
Loss of appetite	Not	664 (88.77)	417 (83.57)	1.00	-
	Yes	84 (11.23)	82 (16.43)	1.55 (1.12–2.16)	.008
Fatigue	Not	624 (83.42)	345 (69.14)	1.00	-
	Yes	124 (16.58)	154 (30.86)	2.25 (1.71–2.94)	<.001
History of previous tuberculosis treatment	Not	561 (75.00)	316 (63.33)	1.00	-
	Yes	187 (25.00)	183 (36.67)	1.75 (1.37–2.23)	<.001
Lineage	Other	33 (4.41)	28 (5.61)	1.00	-
	Lineage2	497 (66.44)	316 (63.33)	0.75 (0.44–1.26)	.280
	Lineage4	218 (29.14)	155 (31.06)	0.84 (0.49–1.44)	.524
Ethnicity	Han	517 (69.12)	323 (64.73)	1.00	-
	Zhuang	221 (29.55)	160 (32.06)	1.16 (0.91–1.48)	.241
	Other	10 (1.34)	16 (3.21)	2.56 (1.15–5.71)	.022
Occupation	Other	41 (5.48)	37 (7.41)	1.00	-
	Farmer	571 (76.34)	398 (79.76)	0.77 (0.49–1.23)	.274
	Housework and unemployment	123 (16.44)	63 (12.63)	0.57 (0.33–0.97)	.039
	Student	13 (1.74)	1 (0.20)	0.09 (0.01–0.68)	.020
PZA	Wild-type*	722 (96.52)	481 (96.39)	1.00	-
	pnca.p	21 (2.81)	13 (2.61)	0.93 (0.46–1.87)	.837
	pnca.c	5 (0.67)	5 (1.00)	1.50 (0.43–5.21)	.522
RFP	Wild-type*	623 (83.29)	381 (76.35)	1.00	-
	rpoB_p.Ser450	51 (6.82)	58 (11.62)	1.86 (1.25–2.77)	.002
	rpoB_p.His445	29 (3.88)	18 (3.61)	1.01 (0.56–1.85)	.962
	rpoB_p.Leu430	6 (0.80)	9 (1.80)	2.45 (0.87–6.95)	.091
	rpoB_.Leu452	10 (1.34)	4 (0.80)	0.65 (0.20–2.10)	.476
	Double mutation	15 (2.01)	19 (3.81)	2.07 (1.04–4.12)	.038
	Other	14 (1.87)	10 (2.00)	1.17 (0.51–2.66)	.711
INH	Wild-type*	583 (77.94)	372 (74.55)	1.00	-
	katG_p.Ser315	116 (15.51)	90 (18.04)	1.22 (0.90–1.65)	.208
	inhA	8 (1.07)	7 (1.40)	1.37 (0.49–3.81)	.545
	fabG1	9 (1.20)	1 (0.20)	0.17 (0.02–1.38)	.098
	aphC	6 (0.80)	3 (0.60)	0.78 (0.19–3.15)	.731
	Double mutation	12 (1.60)	13 (2.61)	1.70 (0.77–3.76)	.192
	Other	14 (1.87)	13 (2.61)	1.46 (0.68–3.13)	.337
EMB	Wild-type*	689 (92.11)	448 (89.78)	1.00	-
	embB_p.Met306	27 (3.61)	23 (4.61)	1.31 (0.74–2.31)	.352
	embB_p	8 (1.07)	13 (2.61)	2.50 (1.03–6.08)	.043
	embA_c	20 (2.67)	12 (2.40)	0.92 (0.45–1.91)	.828
	Double mutation	4 (0.53)	3 (0.60)	1.15 (0.26–5.18)	.852
S	Wild-type*	670 (89.57)	448 (89.78)	1.00	-
	rpsL_p.Lys43	41 (5.48)	18 (3.61)	0.66 (0.37–1.16)	.146
	rrs.n. 514	13 (1.74)	6 (1.20)	0.69 (0.26–1.83)	.456
	rrs.n	10 (1.34)	15 (3.01)	2.24 (1.00–5.04)	.050
	Double mutation	4 (0.53)	1 (0.20)	0.37 (0.04–3.36)	.380
	rpsL_Other	10 (1.34)	11 (2.20)	1.65 (0.69–3.91)	.259
Educational level	Illiterate person	68 (9.09)	39 (7.82)	1.00	-
	Elementary school	262 (35.03)	211 (42.28)	1.40 (0.91–2.17)	.125
	Secondary school	302 (40.37)	191 (38.28)	1.10 (0.71–1.70)	.658
	High school	91 (12.17)	46 (9.22)	0.88 (0.52–1.50)	.640
	University	25 (3.34)	12 (2.40)	0.84 (0.38–1.85)	.660

Age variables are described as “X ± S,” while other variables are expressed as “number (percentage/%) [*n* (%)].”

Wild-type*: It is defined as the absence of detected mutations in the target genes/loci screened.

**Figure 2.**
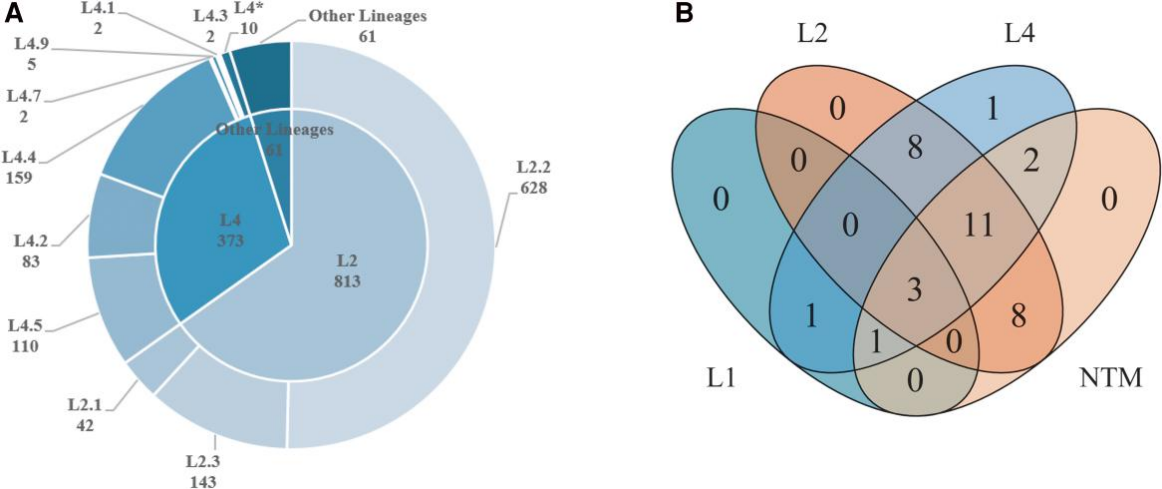
Lineage distribution and mixed-lineage infection characteristics. *A*, Pie chart of different subtypes of the pedigree. Note: Lineage 4*: These strains could not be classified into specific subtypes; The numbers in the figure represent the strain count for each lineage; “Other-Lineages” refers to the group comprising lineage L1, L3, L6, La1.7, and mixed-lineage infections. *B*, Mixed-lineage infections among lineage groups.

The strain lineages identified in this study exhibited substantial diversity. The majority were classified as Lineage 2 (L2, *n* = 813/1247, 65.20%) and Lineage 4 (L4, *n* = 373/1247, 29.91%), with a small number belonging to L1 (*n* = 20), *Non-tuberculous mycobacteria* (NTM) (*n* = 5), and L3 (*n* = 1) (refer to Figure 2*A*). Additionally, 35 strains (2.81%) showed evidence of mixed-lineage infections. Among these, coinfection with L2 + L4 + NTM was most common (*n* = 11), followed by L2 + L4 (*n* = 8) and L2 + NTM (*n* = 8) (detailed in Figure 2*B*). Given the predominance of L2 and L4, the resistance profiles to RFP, INH, PZA, EMB, and S were compared for these 2 major lineages in Table 2. The results demonstrated statistically significant differences in RFP and S resistance between L2 and L4 (*χ*^2^ = 5.807, 27.045, *P* < .05), whereas no significant differences were observed for INH, PZA, or EMB resistance across lineages. Among the strains, 132 cases were MDR in L2 and 39 in L4, indicating statistically significant differences in MDR between lineages (*χ*^2^ = 6.924, *P* = .009).

**Table 2.** Comparison of Drug Resistance Between Lineage L2 and L4 Strains

Drug Resistance	Total	L2 (n, %)	L4 (*n*, %)	*χ* ^2^	*P* Value
RFP				5.807	.016
Resistance	240	180 (22.14)	60 (16.09)		
Sensitive	946	633 (77.86)	313 (83.91)		
INH				3.263	.071
Resistance	274	200 (24.60)	74 (19.84)		
Sensitive	912	613 (75.40)	299 (80.16)		
PZA				2.563	.109
Resistance	44	35 (4.30)	9 (2.41)		
Sensitive	1142	778 (95.70)	364 (97.59)		
EMB				1.570	.238
Resistance	90	67 (8.24)	23 (6.17)		
Sensitive	1096	746 (91.76)	350 (93.83)		
S				27.045	<.001
Resistance	117	105 (12.91)	12 (3.22)		
Sensitive	1069	708 (87.08)	361 (96.78)		

### Comparison of Gene Sequencing Results With pDST

As shown in Table 3, with sequencing as the reference standard, pDST demonstrated a sensitivity of 62.33% (95% CI: 56.64%–67.78%) and a specificity of 95.81% (95% CI: 94.27%–96.95%) for detecting INH resistance, with a positive predictive value (PPV) of 81.98% (95% CI: 76.44%–86.46%) and a negative predictive value (NPV) of 89.27% (95% CI: 87.26%–91.06%). The overall agreement between the 2 methods was 87.97%, with a Youden's index of 0.581 and a *κ* value of 0.634, showed moderate consistency, and the difference between them was statistically significant (*χ*^2^ = 32.667, *P* < .001).

**Table 3.** Concordance Between Genotypic and Phenotypic Assays for Detecting INH Resistance

PDST	Genetic Sequencing		Total
	Resistance	Sensitive	
Resistance	182	40	222
Sensitive	110	915	1025
Total	292	955	1247

In Table 4, with sequencing as the reference standard, pDST demonstrated a sensitivity of 78.60% (95% CI: 73.12%–83.33%) and a specificity of 96.61% (95% CI: 95.43%–97.52%) for detecting RFP resistance, with a PPV of 84.89% (95% CI: 79.70%–88.99%) and a NPV of 94.91% (95% CI: 93.37%–96.09%). The overall agreement between the 2 methods was 93.10%, with a Youden's index of 0.752 and a *κ* value of 0.774, showed substantial agreement, and the difference between them was not statistically significant (*χ*^2^ = 3.767, *P* = .052).

**Table 4.** Concordance Between Genotypic and Phenotypic Assays for Detecting RFP Resistance

pDST	Genetic Sequencing		Total
	Resistance	Sensitive	
Resistance	191	34	225
Sensitive	52	970	1022
Total	243	1004	1247

### Genetic Mutations

**Figure 3.**
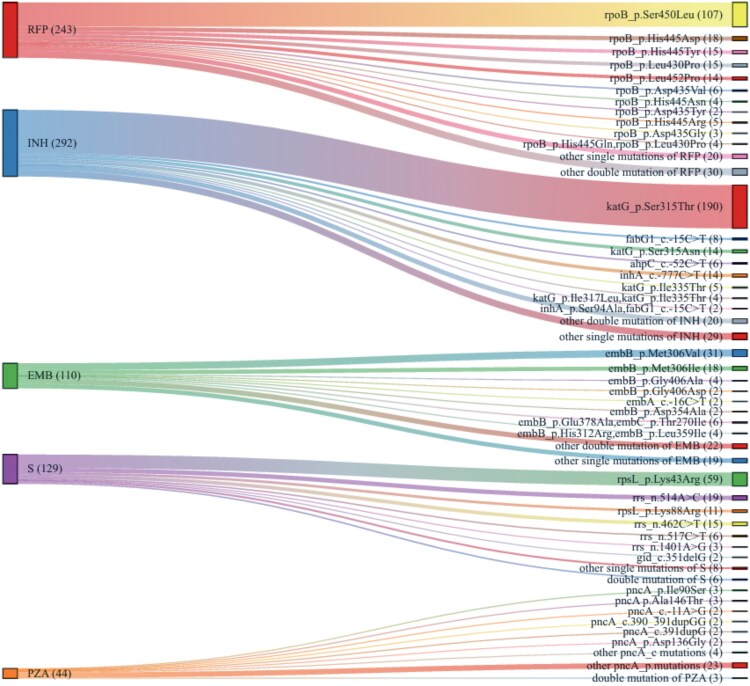
Sankey diagram of drug-gene loci based on mutation frequency.

WGS was performed on 1247 bacterial strains, and the results showed. A total of 243 RFP-resistant strains were detected, with a resistance detection rate of 19.49%. All strains exhibited mutations in the rpoB gene, including 241 missense mutations and 2 insertion mutations (rpoB_c.1291_1293dupAGC, rpoB_c.1329_1331dupGAC). The predominant missense mutations were Ser450Leu (107/243), His445Asp (18/243), His445Tyr (15/243), Leu430Pro (15/243), Leu452Pro (14/243), and Asp435Val (6/243). Four strains harbored the double mutation rpoB_p.His445Gln and rpoB_p. Leu430Pro, etc. Among these double mutants, 4 strains also carried mutations in the rpoC gene; additionally, 292 INH-resistant strains were identified, with a resistance detection rate of 23.42%. The mutated genes included katG, fabG1, inhA, and ahpC, with the most frequent mutation being katG_p.Ser315Thr (190/292), followed by inhA_c.-777C>T (14/292), katG_p.Ser315Asn (14/292), fabG1_c.-15C>T (8/292), and ahpC_c.-52C>T (6/292), katG_p.Ile335Thr (5/292), and 4 strains exhibited dual mutations of katG_p.Ile317Leu and katG_p.Ile335Thr, etc. A total of 44 PZA-resistant strains were detected, with a resistance detection rate of 3.53%. Mutations in the pncA gene, including pncA_p and pncA_c, were observed, with multiple types, among which Ala146Thr (3/44) and Ile90Ser (3/44) were most common; 110 EMB-resistant strains were identified, with a resistance detection rate of 8.82%, predominantly involving mutations in embB and embA. The most frequent mutation was embB_p.Met306Val (31/110), followed by embB_p.Met306Ile (18/110), embB_p.Gly406Ala (4/110) was observed, with 6 strains exhibiting dual mutations of embB_p.Glu378Ala and embC_p.Thr270Ile, and 4 strains showing dual mutations of embB_p.His312Arg and embB_p.Leu359Ile. A total of 129 S-resistant strains were detected, with a resistance detection rate of 10.34%. Mutations in the rpsL, rrs, and gid genes were observed, with rpsL_p.Lys43Arg (59/129) being the predominant mutation, rrs_n.514A>C (19/129), rrs_n.462C>T (15/129), rpsL_p.Lys88Arg (11/129), and rrs_n.517C>T (6/129), with 6 strains exhibiting biallelic mutations, etc. Detailed information on the drug-related gene mutations is provided in Figure 3 and Supplementary Table 3.

We identified 7 MDR profiles resulting from polygenic mutations: RFP + INH (*n* = 172, 13.79%), RFP + INH + PZA (*n* = 33, 2.65%), RFP + INH + EMB (n = 69, 5.53%), RFP + INH + S (*n* = 61, 4.89%), RFP + INH + PZA + EMB (*n* = 27, 2.17%), RFP + INH + PZA + S (*n* = 25, 2.00%), and RFP + INH + PZA + EMB + S (*n* = 21, 1.68%). Specific mutations and other mutation combinations are detailed in Supplementary Table 4.

### Factor Selection for the Predictive Model

**Figure 4.**
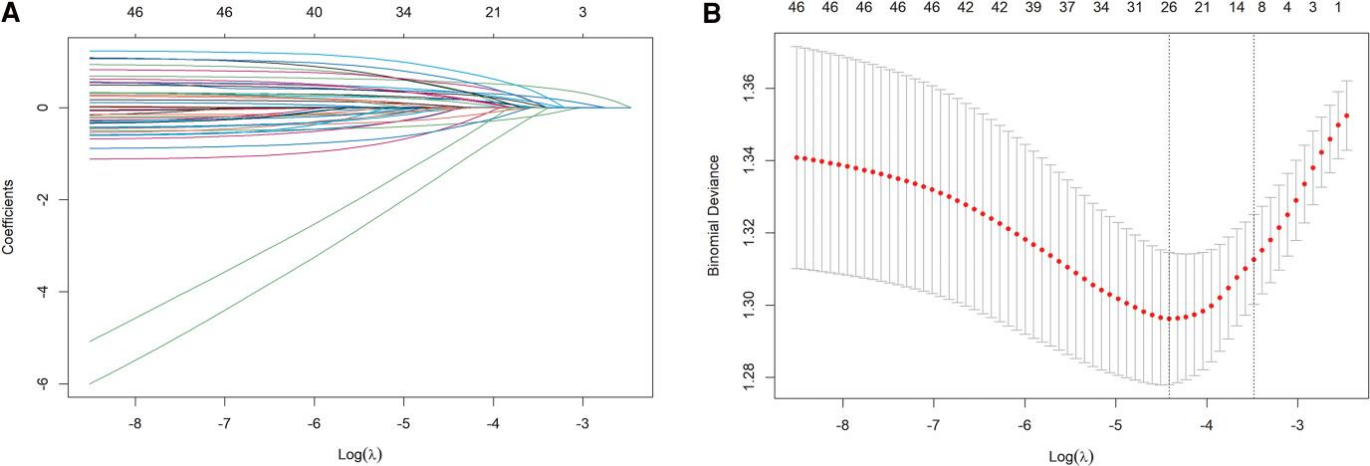
Lasso Regression: coefficient paths and cross-validation curve. *A*, Plot of the Lasso coefficient profiles. *B*, Tuning parameter (λ) selection cross-validation error curve.

The Lasso regression was performed with the grouping variable as the dependent variable and all other variables as independent variables. The optimal lambda value was selected through 10-fold CV. To obtain a parsimonious model, lambda.1se (λ = 0.03064848) was chosen as the final penalty parameter. The coefficient path plot and CV curve are shown in Figure 4, respectively. The Lasso regression reduced the initial set of 24 variables to 10, which included fatigue, fever, history of TB treatment, gender, age, RFP, INH, occupation, S, and ethnicity. To further control the influence of confounding factors, multiple logistic regression analysis was conducted on the variables selected by the Lasso regression analysis mentioned above. Eventually, gender, age, fever, fatigue, previous history of TB treatment, rpoB_p.Ser450 and other ethnicities were included as characteristic factors (*P* < .05) (as shown in Table 5).

**Table 5.** Multivariable Logistic Regression Analysis Incorporating Variables Selected by Lasso

Variables	Crude OR (95% CI)	Adj. OR (95% CI)	*P* (Wald's Test)	*P* (LR-test)
Gender				.037
Male (reference)				
Female	0.67 (0.49, 0.91)	0.71 (0.51, 0.98)	.039	
Age	1.02 (1.01, 1.03)	1.01 (1.01, 1.02)	<.001	<.001
Fever				.009
Not (reference)				
Yes	1.59 (1.19, 2.13)	1.5 (1.11, 2.04)	.009	
Fatigue				<.001
Not (reference)				
Yes	2.25 (1.71, 2.94)	1.91 (1.44, 2.53)	<.001	
Tuberculosis history				.003
Not (reference)				
Yes	1.74 (1.36, 2.22)	1.47 (1.14, 1.91)	.003	
rpoB.Ser450				.02
Not (reference)				
Yes	1.8 (1.21, 2.67)	1.63 (1.08, 2.47)	.02	
Occupation.Student				.068
Not (reference)				
Yes	0.11 (0.01, 0.87)	0.19 (0.02, 1.66)	.134	
S.rrs.n.other				.05
Not (reference)				
Yes	2.29 (1.02, 5.13)	2.28 (0.99, 5.25)	.052	
Ethnicity.Other				.009
Not (reference)				
Yes	2.44 (1.1, 5.43)	2.94 (1.29, 6.73)	.011	

### Comparative Analysis of Multiple ML Models

**Figure 5.**
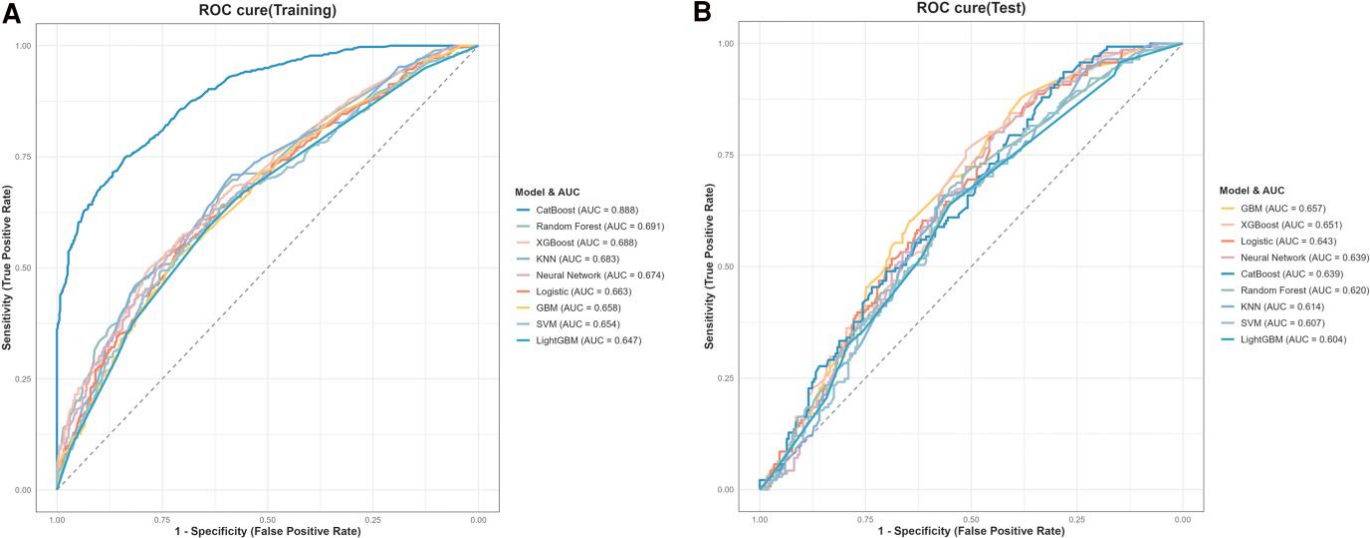
Summary of AUC for training and test sets of each model. *A*, Training sets ROC and AUC. *B*, Test sets ROC and AUC.

**Figure 6.**
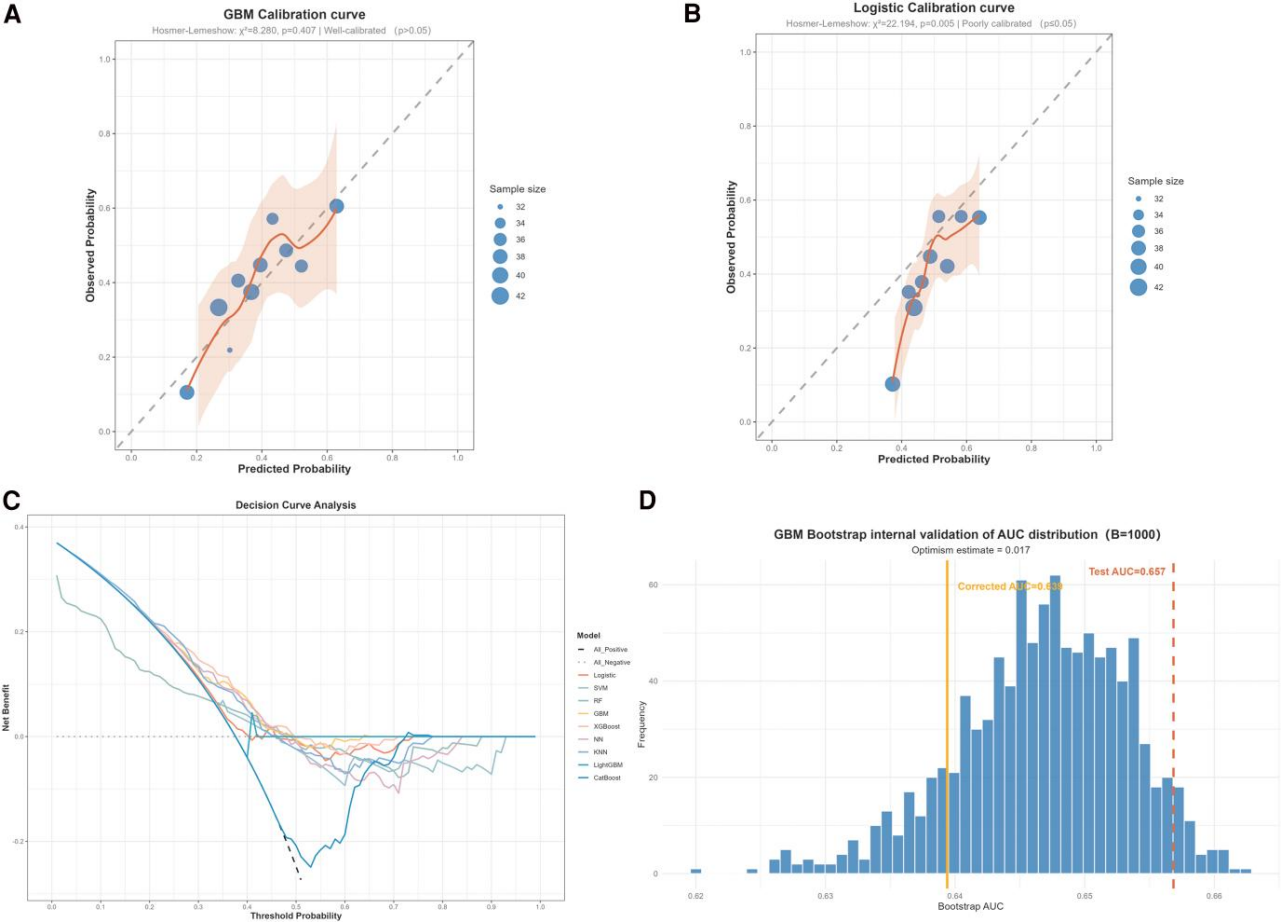
Comprehensive Validation of the GBM Prediction Model. *A*, GBM calibration curve. *B*, Logistic calibration curve. *C*, Decision curve analysis comparing net clinical benefit. *D*, GBM Bootstrap internal validation.

The variables screened by Lasso regression analysis were trained using 9 ML models. All models were evaluated using 5-fold CV, and the AUC was used to assess model performance. The results showed that the CatBoost model achieved the highest AUC value of 0.888 on the training set but had a lower AUC of 0.639 on the test set, indicating a risk of overfitting. The GBM model performed well with an AUC of 0.658 on the training set, an AUC of 0.657 on the test set, and an F1 score of 0.605. The Logistic model had a lower AUC of 0.643 on the test set but a lower F1 score of 0.586, as shown in Table 6 and Figure 5. DeLong's test was performed on the Logistic and GBM models, revealing no significant difference in AUC (*P* > .05). And the Hosmer–Lemeshow test showed: *χ*^2^ = 8.280, *P* = .407, the predictions of the GBM model were in good agreement with the observed results (Figure 6*A* and 6*B*, other models are detailed in Supplementary Figure 2). DCA revealed that the GBM model yielded superior net benefit over conventional logistic regression across a clinically meaningful threshold probability range of 10%–45% (Figure 6*C*). After conducting internal validation on the model, the optimism-corrected AUC was 0.639 (95% CI: 0.632–0.658) (Figure 6*D*), the fitting effect of GBM was also quite good. Taken together, these findings suggest that the GBM model was the most desirable model for this study and a SHAP analysis was conducted.

**Table 6.** Performance Metrics of the 9 ML Models

Model	AUC^a^	AUC^b^	Accuracy	Sensitivity	Specificity	PPV	NPV	F1
GBM	0.658	0.657	0.568	0.879	0.380	0.461	0.840	0.605
XGBoost	0.688	0.651	0.605	0.759	0.513	0.484	0.779	0.591
Logistic	0.663	0.643	0.581	0.787	0.457	0.466	0.781	0.586
NN	0.674	0.639	0.579	0.794	0.449	0.465	0.784	0.586
CatBoost	0.888	0.639	0.528	0.936	0.282	0.440	0.880	0.599
RF	0.691	0.620	0.597	0.674	0.551	0.475	0.737	0.557
KNN	0.683	0.614	0.600	0.660	0.564	0.477	0.733	0.554
SVM	0.654	0.607	0.592	0.624	0.573	0.468	0.717	0.535
LightGBM	0.647	0.604	0.584	0.638	0.551	0.462	0.717	0.536

^a^AUC: AUC value for the training set.

^b^AUC: AUC value for the test set.

### Model Interpretability

**Figure 7.**
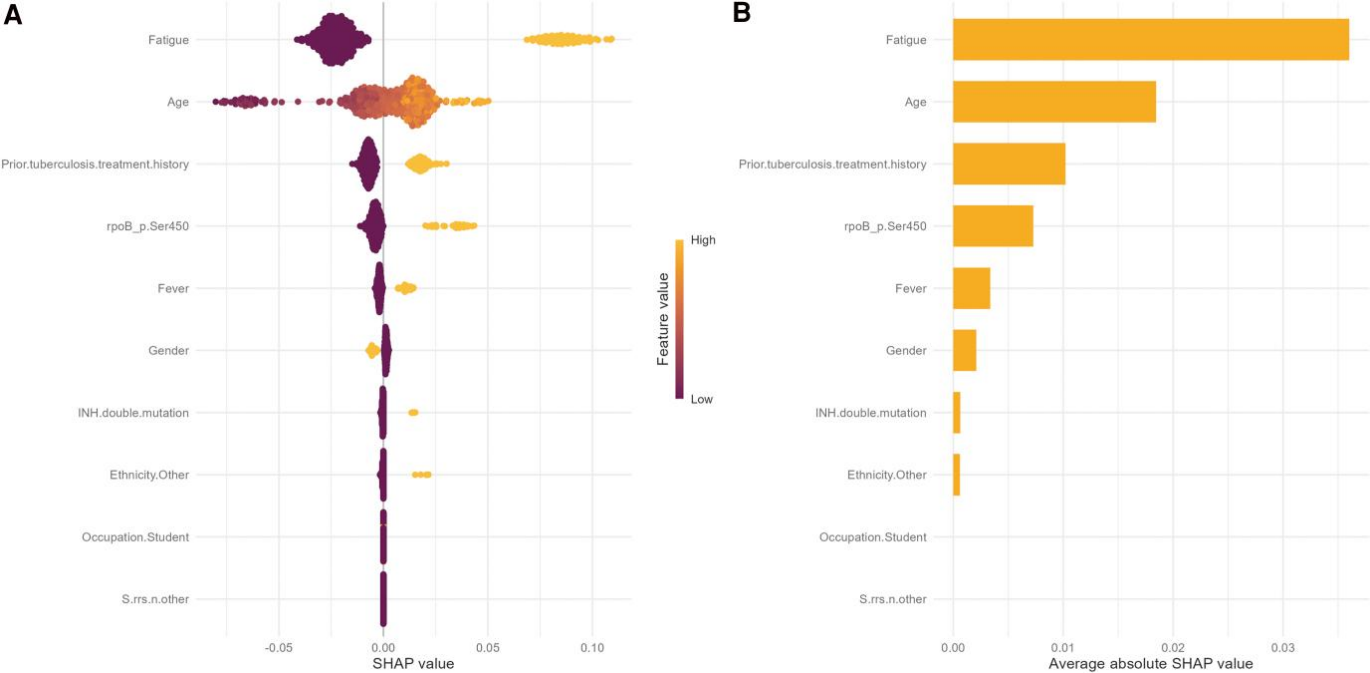
SHAP explanation model and variable importance. *A*, Attributes of characteristics in SHAP. *B*, Feature importance ranking as indicated by SHAP.

**Figure 8.**
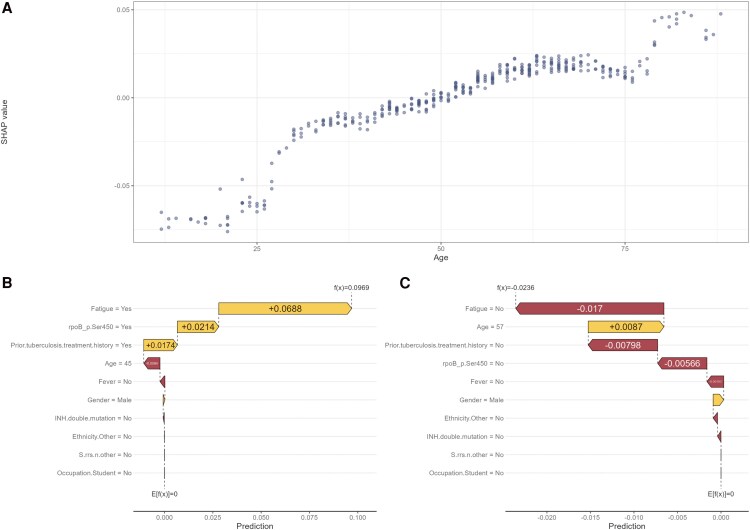
Individual prediction explanation using SHAP waterfall Chart. *A*, Dependency graph of the age variable. *B*, SHAP explanation for the high predicted risk case. *C*, SHAP explanation for the low predicted risk case.

To visually illustrate the selected variables, SHAP was employed to explain how these variables contribute to the model's prediction of the occurrence of severe cavities. SHAP reflects its importance and direction of association in the models predictions, each feature significance line showcases all patient attributions for the outcome, represented by differently colored dots: yellow dots indicate high-risk values, while purple dots denote low-risk values. The results indicate that fatigue, older age, history of previous TB treatment, repeated fever, male, and rpoB_p.Ser450 mutation are associated with the occurrence of severe cavities, as shown in Figure 7. To evaluate the robustness of the SHAP results, we performed a bootstrap stability analysis with 1000 resamples, and the validation results (Supplementary Figure 3) confirmed the stability of the SHAP analysis. Two single-sample waterfall plots were used to enhance the interpretability of the model. The SHAP waterfall plot reveals the predictive contributions of various clinical factors to the predicted probability of severe cavitation in patients. In Figure 8*A* illustrates the dependence of SHAP values on age characteristics for the prediction results, demonstrating that different age groups have varying predictive power in the model. In Figure 8*B*, among the 45-year-old patient, age serves as the primary protective factor, reducing the predicted probability of severe cavitation, whereas the patient's fatigue, rpoB_p.Ser450 mutation, and history of previous TB treatment increase the predicted probability, with quantitative effects of 0.0688, 0.0214, and 0.0174, respectively. Conversely, in Figure 8*C*, 57-year-old age and male sex increase the predicted probability of disease occurrence, while the absence of fatigue, history of previous TB treatment, and rpoB_p.Ser450 mutation decrease the predicted probability. In addition, SHAP dependence plot illustrates the relationship between a single feature and its contribution to the model's output, with each point representing a patient. The results show that age and fatigue have a synergistic effect: an increase in age not only elevates SHAP values but also leads the model to predict a higher probability of severe cavities. In contrast, the rpoB_p.Ser450 mutation shows an antagonistic interaction with fatigue (Supplementary Figure 4).

## DISCUSSION

This study enrolled a total of 1247 patients, predominantly male and farmers, with L2 and L4 dominating the bacterial spectrum, demonstrating significant lineage diversity. The most common drug-resistance gene mutations were rpoB_p.Ser450Leu, katG_p.Ser315Thr, embB_p.Met306Val, and rpsL_p.Lys43Arg, mutations associated with PZA resistance exhibited greater heterogeneity. This study systematically characterized the predominant lineages and drug-resistance mutation profiles of MTB in patients with cavitary TB, providing critical molecular epidemiological data for local drug-resistance surveillance. Further analysis using Lasso regression and 9 ML models identified features associated with severe cavitary formation, revealing that fatigue, fever, advanced age, history of previous TB treatment, male, and rpoB_p.Ser450 mutation were positively associated with severe cavitary PTB.

Analyzing the epidemiology, drug resistance, and transmission characteristics of MTB isolates is crucial for implementing more effective TB prevention and control strategies. Studies have found that the L1 and L3 are primarily prevalent in East Africa and Southeast Asia [24, 25]. Given the relatively low number of L1 and L3 identified in our study, further research is needed to confirm these observations. According to previous studies, the Beijing lineage is the predominant lineage in China, with provincial variations ranging from 44% to 93% [26, 27]. Our findings revealed that 65.20% of the strains belonged to L2, a proportion similar to that reported in Wuzhou (69.8%) [28]. Notably, 3.37% of the isolates were identified as the proto-Beijing genotype (L2.1), which is uncommon in other regions of China and represents a distinctive feature of MTB strains in Guangxi. Geographically, Guangxi shares a border with Vietnam, where L2.1 strains have also been reported [29]. These findings imply a potential risk of cross—border transmission in this area; however, further investigation is necessary for verification.

We conducted a consistency comparison between RFP and NIH drug-resistance phenotypes and WGS-based genotypes, finding moderate consistency for INH (*κ* = 0.634) with statistically significant differences in consistency testing, suggesting discordant results between the 2 methods. This observed discordance may be attributed to several factors, including the heterogeneous drug resistance in MTB samples. Heterogeneous drug-resistance refers to the coexistence of sensitive and resistant strains in clinical samples from a single TB patient, primarily caused by coinfection of sensitive and resistant strains or resistant mutations in certain subpopulations of sensitive strains [30]. In cases of discordance between phenotypic and genotypic drug-resistance profiles in clinical MTB isolates, heteroresistance is considered a primary contributing factor [31, 32]. Excluding the impact of heteroresistance on genotypic assays could substantially improve the concordance between genotype and phenotype. Previous studies have shown that drug-resistance heterogeneity in some patients is associated with mixed infections [33, 34]. Patients with mixed infections are at increased risk of developing such heterogeneity, because the 2 strains can have distinct drug-resistance profiles [35]. And it may also be caused by novel mutations not included in the drug-resistance mutation catalogs (phenotypic resistant, genotypic sensitive), by heteroresistance resulting from low-frequency minority mutations (phenotypic sensitive, genotypic resistant), or by low-level resistance mutations undetected by phenotypic methods (phenotypic sensitive, genotypic resistant) [36, 37]. Additionally, there is also the possibility that the incorrect interpretation was caused by human factors during the phenotypic drug sensitivity test.

RFP is a highly effective first-line anti-TB agent. It exerts its bactericidal activity by binding to the β-subunit of bacterial RNA polymerase (RNAP), thereby blocking the elongation of nascent RNA transcripts and ultimately inhibiting transcription [38]. Compensatory mutations resistant to RFP may occur in the α subunit (rpoA), β′ subunit (rpoC), or even the rpoB subunit itself, thereby mitigating the adverse effects on the host [39]. MTB primarily acquires RFP resistance through rpoB mutations, with over 95% of these mutations located within an 81-bp RFP resistance determining region (RRDR) [40]. To overcome the fitness cost, secondary mutations are selected to improve or promote the target protein itself or alternative pathways with similar functions. These compensatory mutations typically occur in genes encoding the same protein or genes involved in similar metabolic pathways, thereby reducing the harmful effects of the specific functions impaired by the resistant mutations [41]. Such mutations are closely associated with increased transmission of resistant strains [42]. In this study, RFP resistance was primarily mediated by mutations at codons 450, 445, 430, and 435 of the rpoB gene, demonstrating notable polymorphism. Among these, the p. Ser450Leu substitution was the most frequent, accounting for 44.03% of cases, which is consistent with the results of Ye et al [43] in Guangxi, and also similar to the domestic frequency (45.76%) reported by Li et al [44]. Furthermore, Vadakunnel et al [45] also reported Ser450 as the most common rpoB mutation (54.7%) in RR-TB patients in India, which is in agreement with our results. It is noteworthy that we identified 4 strains carrying compensatory mutations in the rpoC gene (p.Ile491Thr, p.Phe452Ser, p.Leu527Val, and p.Gly332Arg), all of which also harbored the canonical resistance mutation rpoB_p.Ser450Leu. Moreover, 3 of these strains concurrently exhibited mutations in the katG gene, which is consistent with the result that at least one compensatory mutation was present in MDR strains [46]. Similarly, 4 genes were codetectable for INH resistance mutations: katG, fabG1, ahpC, and inhA, with katG mutations being predominant, the primary mutations occurred at the Ser315Thr and Ser315Asn sites, consistent with previous study [47]. The embB_p.Met306Val mutation was the most prevalent among EMB-resistant isolates in our study, which aligns with prior reports [48], followed by embB_p.Met306Ile. The mutation types resistant to S are predominantly rpsL_p.Lys43Arg, which aligns with results from southern China [49]. In contrast, mutations resistant to PZA are scattered across the pncA gene and promoter, with pncA. Ile90Ser being the most frequent (unlike the commonly observed pncA_c.A-11G in our study) [48]. The observed differences may be attributed to the local transmission of drug-resistant strains and region-specific evolutionary pressures. The above findings indicate that although the predominant gene mutation types among drug-resistant strains are largely similar across different regions, the specific mutation loci display notable diversity. These observations underscore the need for further targeted research to generate robust evidence in support of precision strategies for TB treatment and control.

Lasso regression is a model based on penalty functions that effectively compresses regression coefficients. Using Lasso regression can screen out the most representative predictors and address the issue of biased estimation in data with multicollinearity [50]. In this study, after applying Lasso regression and fitting several ML models, the CatBoost model exhibits insufficient stability due to a marked discrepancy in AUC between the training and test sets. By contrast, the GBM model maintains high consistency across both sets, achieving a superior balance between training and test performance and better generalization than CatBoost. In addition, calibration plots and the Hosmer–Lemeshow test confirm good calibration of the GBM model. The GBM model also provides higher net clinical benefit than logistic regression, and internal validation confirms its robust fit. Therefore, we ultimately selected GBM for analysis and used the SHAP method to interpret the model, identifying 6 key variables: fatigue, fever, older age, history of previous TB treatment, male, and rpoB_p.Ser450 mutation as features positively associated with severe cavitary PTB. In our study, the mean age of enrolled patients was 53 years, older age was positively associated with disease progression, which aligns with findings from prior studies [51]. This may be attributed to several factors: physiologically, declining respiratory defense mechanisms and reduced immune competence in older adults diminish the ability to contain MTB infection. Socioeconomically, a majority of patients resided in rural areas with limited financial resources and lower health literacy, which likely contributed to delays in seeking care upon symptom onset, thereby increasing the risk of disease progression. As observed in global studies, we also found that males have a higher risk of adverse outcomes, consistent with previous finding [52], social behavioral factors such as alcohol consumption, smoking, and poor treatment adherence may explain this gender difference. Typical symptoms commonly observed in patients with PTB include chronic cough, fever, night sweats, fatigue, and weight loss [53]. Our study found that fever and fatigue are risk factors for severe cavitation. Patients with different stages of TB exhibit varying clinical symptoms, the exacerbation of fever and cough may indicate disease progression, as compromised immune function affects the cytokine level of IFN-γ secreted by T lymphocytes [54]. Coupled with the inherently weaker immune status in elderly patients, this may lead to the progression of tuberculous cavitation to a more severe stage. The cytokines interleukin (IL)-1, IL-6, and tumor necrosis factor (TNF)-alpha induce increases in body temperature via direct and indirect actions on the brain and are believed to act as endogenous pyrogens [55]. During TB infection, pro-inflammatory (TNF-α, IL-1, IL-6, and IL-18) and anti-inflammatory (IL-10) cytokines are predominantly produced by *MTB*-infected pulmonary macrophages. These cytokines serve as essential immune mediators for host control of the infection, but when overexpressed, they can also drive systemic inflammatory responses and cavity formation [56]. Maseko et al found that higher Interleukin-6 plasma levels (aRR = 1.405, 95% CI 1.079–1.829) were associated with an increased risk of lung cavitation in patients with drug-resistant TB [57]. In line with this, our study showed that fever, as a surrogate marker of systemic inflammation, was also associated with an elevated risk of pulmonary cavitation. Previous studies have shown that treatment delay [16], bacterial burden [58], smoking [59], alcohol use [60], nutritional status [61], and socioeconomic status [62] are all associated with cavitation in PTB.

MDR of MTB strains is a significant factor related to cavity formation. In MDR cases, MTB develops resistance to anti-TB drugs, leading to chronic progressive disease and the development of pulmonary cavities in patients [63]. Previous studies have demonstrated that the presence of pulmonary cavities is closely associated with drug resistance during treatment, and the interaction between pulmonary cavities and drug resistance may accelerate the formation and expansion of cavities [64]. Zhao et al [65] found that the presence of pulmonary cavities was independently associated with MDR, with an OR of 2.017 for cavitary versus noncavitary patients. Previous studies have seldom investigated the association between drug-resistant gene mutations and pulmonary cavities. In our study, the rpoB_p.Ser450 mutation was positively associated with severe cavitary PTB. Kim et al [66] found that a history of previous TB treatment is associated with the occurrence of cavitary TB, which is consistent with our results. The population included in this study originated from Guangxi region, mainly consisting of elderly male farmers. The characteristics of this population were consistent with those of the high-risk group (males, elderly) reported by Li et al [51] in their study on cavity absorption in Tianjin. Additionally, Zhang et al [64] in Beijing found that multidrug resistance was a risk factor for cavities. And Chen et al [67] analysis of RFP resistance characteristics in Guizhou region also showed that the Ser450Leu mutation in the rpoB gene was the most common SNP. These cross-regional research results have good consistency with the findings of this study.

Although the discrimination ability of this model is at a moderate level, it may still provide certain auxiliary value for risk assessment in specific clinical scenarios—especially when the model results are combined with clinical judgment (rather than completely replacing clinical decisions). It should be noted that predicting the formation of PTB cavities is inherently challenging to some extent: the occurrence and development of cavities are not only influenced by the virulence and drug-resistance mutations of MTB, but also related to various factors such as the host's immune status, age, nutritional status, smoking history, previous treatment history, and comorbidities. Therefore, the prediction results provided by this model can still offer certain references for the identification of populations with a higher predicted probability. For the key predictive variables identified in this study model (rpoB_p.Ser450), given the prolonged duration of WGS and its detection cycle often exceeding the clinical diagnosis window, there is an urgent need to develop rapid detection tools based on these molecular markers for clinical TB specialty clinics. Such tools could include allele-specific PCR kits targeting the rpoB_p.Ser450 site mutation or rapid detection reagents for site mutations. Integrated with the clinical risk profile derived from this study (fatigue, male, older age, history of previous TB treatment, and fever), these rapid tools can assist clinicians (particularly primary care physicians in resource-limited settings) in early identification of patients prone to severe cavitary TB, thereby enabling early warning and proactive intervention.

This study has the following limitations: First, this single-center study could not perform external validation, and the model's discrimination needs improvement. We plan prospective, multicenter external validation and model refinement. Second, some host factors were not included—such as CD4+/CD8+ T-cell counts, smoking/alcohol use, BMI, treatment delay, smear/culture burden, and radiographic extent of cavitation. Third, although rpoB_p.Ser450 was associated with severe cavitation, causality cannot be inferred from observational data.

## CONCLUSION

The rpoB_p.Ser450 mutation is linked to severe cavitary PTB, but clinical and population studies are still needed to confirm this association. Therefore, new tools based on clinical indicators and biomarkers are needed to achieve early warning and timely intervention for the risk of severe cavitation.

## Supplementary Material

ofag328_Supplementary_Data

## Supplementary Data

Supplementary materials are available at *Open Forum Infectious Diseases* online. Consisting of data provided by the authors to benefit the reader, the posted materials are not copyedited and are the sole responsibility of the authors, so questions or comments should be addressed to the corresponding author.
