# Supplementary material for: Analysis of Drug Resistance Characteristics and Risk Factors of Cavitary Tuberculosis Based on Whole Genome Sequencing and Machine Learning
Source: Open Forum Infect Dis. 2026 Jun 3;13(6):ofag328. doi: 10.1093/ofid/ofag328 (PMC13270246; doi:10.1093/ofid/ofag328)
Supplement: ofag328_Supplementary_Data [file ofag328_supplementary_data.docx]

**Supplementary Method**

Strict quality control (QC) measures were implemented throughout the entire pDST process to ensure the reliability and accuracy of the experimental results, with detailed procedures described as follows:

**Control strains**: Two standard reference strains were incorporated into each test batch for quality control purposes, including the drug-susceptible Mycobacterium tuberculosis strain H37Rv (ATCC 27294) and the multidrug-resistant (MDR) reference strain H37Rv-RIF-INH (ATCC 35822). The H37Rv strain was anticipated to exhibit susceptibility to all tested first-line anti-tuberculosis drugs, whereas the H37Rv-RIF-INH strain was expected to be resistant to INH and RFP, but susceptible to EMB, PZA, and S. Test results of clinical isolates were considered valid only when the quality control strains displayed the expected susceptibility/resistance phenotypes; otherwise, the entire test batch was repeated.

**Criteria for resistance/susceptibility calls:** The interpretation of susceptibility and resistance phenotypes was strictly in accordance with the guidelines for solid-medium pDST of Mycobacterium tuberculosis recommended by the World Health Organization (WHO). Specifically, for INH, RFP, EMB, and S, a strain was classified as resistant if the number of colonies grown on the drug-containing medium accounted for ≥1% of that on the drug-free control medium; otherwise, the strain was deemed susceptible. For PZA, given its unique mechanism of action, any visible colonial growth on the PZA-containing medium indicated resistance, while the absence of colonial growth indicated susceptibility, which was consistent with the instructions provided by the medium manufacturer (Zhuhai BASO Biotechnology Co., Ltd.).

**Handling of borderline growth**: Borderline growth was defined as colonial growth on the drug-containing medium that approached the 1% threshold (i.e., 0.8%–1.2% of the colonial count on the drug-free control medium) for INH, RFP, EMB, and S, or sparse, ambiguous colonial growth on the PZA-containing medium. All samples exhibiting borderline growth were retested in duplicate using freshly prepared drug-containing and drug-free media. If borderline growth persisted in the duplicate tests, the pDST was repeated once more, and the final result was determined based on the majority of the three independent test outcomes.

**Blinding of lab personnel:** personnel performing pDST (inoculation, incubation, colony counting) were blinded to the genomic sequencing data and results during the entire phenotypic testing process. Similarly, those involved in genomic data analysis were unaware of the corresponding pDST phenotypes until all testing was completed. Unblinding was only performed during the final statistical integration of phenotypic and genotypic data

**Range of hyperparameters for machine learning**

For all models, we employed a grid search strategy with 5-fold inner cross-validation on the training set to select optimal hyperparameters. The parameter search spaces for each model were as follows:

**| Model | Key Hyperparameters | Search Range |**

|-------|-------------------|--------------|

| **GBM** | n.trees | [50, 100] |

| | learning_rate | [0.01, 0.05] |

| | max_depth | [1, 2] |

| | n.minobsinnode | [10, 15] |

| | bag.fraction | [0.5] |

| **CatBoost** | iterations | [200, 500, 1000] |

| | learning_rate | [0.01, 0.05, 0.1] |

| | depth | [4, 6, 8, 10] |

| | l2_leaf_reg | [1, 3, 5, 7] |

| | od_type | ["Iter"] |

| | od_wait | [30] |

| **XGBoost** | nrounds | [50,100] |

| | learning_rate | [0.01, 0.05] |

| | max_depth | [2, 3] |

| | eta | [0.01, 0.05] |

| | gamma | [0.1, 0.5] |

| | colsample_bytree | [0.6, 0.8] |

| | min_child_weight | [3,5] |

| | subsample | [0.6, 0.8] |

| |reg_alpha, reg_lambda | [0.1,1] |

| **Random Forest** | mtry| [c(2, 3, 4, 5, round(sqrt(ncol(X_train))))] |

| | ntree | [300] |

| | nodesize | [5] |

| **Logistic** | method | [glmnet] |

| | alpha | [0, 0.5, 1] |

| | lambda | [0.001, 0.005, 0.01, 0.05, 0.1, 0.2, 0.5] |

| **SVM** | sigma | [0.001, 0.005, 0.01, 0.05, 0.1] |

| | C | [0.1, 0.5, 1, 5, 10] |

| | method | [svmRadial] |

| | gamma | [scale, auto] |

| **Neural Network** | method| [nnet] |

| | size | [3, 5, 8, 10] |

| | decay | [0.01, 0.1, 0.5, 1] |

| | MaxNWts | [10000] |

| | maxit | [200] |

| **LightGBM** | num_leaves| [15, 31, 63] |

| | max_depth | [4, 6, 8] |

| | learning_rate | [0.01, 0.05, 0.1] |

| | lambda_l1 | [0.1 ]|

| | lambda_l2 | [1.0] |

| **KNN** | method| [knn] |

| | tuneGrid | [5, 7, 9, 11, 13, 15, 21]

**Table S1. Research variable assignment table**

| Variables | Assignment |
| --- | --- |
| Groups | Mild-to-Moderate Cavitary Group= 0, Severe Cavitary Group= 1 |
| Lineage | Other=1,Lineage 2=2，Lineage 4=3 |
| Treatment | Initial treatment=0，Re-treatment=1 |
| Residence | Town/City=0，Rural area=1 |
| Gender | Male=0，Female=1 |
| RFP | Not=1，rpoB.p.Ser450=2，rpoB.p.His445=3，rpoB.p.Leu430=4，rpoB.p.Leu452=5,Double mutation=6，Other=7 |
| INH | Not=1，katG.p.Ser315=2，inhA=3，fabG1=4，aphC=5， Double mutation=6，Other=7 |
| PZA | Not=1，pncA.p=2，pncA.c=3 |
| EMB | Not=1，embB.p.Met306=2，embB.p.other=3，Double mutation= 4， Other= 5 |
| S | Not=1，rpsL.p.Lys43=2，rrs.n.514=3, rrs.n= 4, Double mutation= 5,Other= 6 |
| Ethnicity | Han= 1, Zhuang= 2,Other= 3 |
| Occupation | Other= 1, Farmer= 2, Housework and unemployment= 3,Student= 4 |
| Educational level | Illiterate person=1，Elementary school=2，Secondary school=3，High school=4，University=5 |
| Diabetes | Not=0，Yes=1 |
| Hepatitis | Not=0，Yes=1 |
| HIV | Not=0，Yes=1 |
| Cough | Not=0，Yes=1 |
| Fever | Not=0，Yes=1 |
| Hemoptysis | Not=0，Yes=1 |
| Chest pain | Not=0，Yes=1 |
| Night sweats | Not=0，Yes=1 |
| Loss of appetite | Not=0，Yes=1 |
| Fatigue | Not=0，Yes=1 |
| Tuberculosis history | Not=0，Yes=1 |

**Table S2. Sensitivity analysis comparing multiple imputation (MI) and complete case analysis (CCA) for factors associated with pulmonary tuberculosis cavitation**

| Variables | Levels | OR (95%CI)(MI) | *P* (MI) | OR (95%CI) (CCA) | *P* (CCA) |
| --- | --- | --- | --- | --- | --- |
| Treatment | Initial treatment | - | - | - | - |
|  | Re-treatment | 0.82(0.39-1.76) | 0.616 | 0.62(0.27-1.41) | 0.252 |
| Age | - | 1.01(1.00-1.02) | 0.006 | 1.01(1.00-1.02) | 0.036 |
| Educational level | Illiterate person | - | - | - | - |
|  | Elementary school | 1.98(1.18-3.31) | 0.010 | 2.16(1.19-3.94) | 0.012 |
|  | Secondary school | 1.67(0.96-2.92) | 0.070 | 1.49(0.78-2.87) | 0.228 |
|  | High school | 1.52(0.80-2.98) | 0.218 | 1.66(0.77-3.58) | 0.197 |
|  | University | 1.53(0.56-4.14) | 0.406 | 2.36(0.75-7.41) | 0.143 |
| Fever | Not | - | - | - | - |
|  | Yes | 1.56(1.13-2.16) | 0.008 | 1.72(1.18-2.50) | 0.005 |
| Chest pain | Not | - | - | - | - |
|  | Yes | 0.78(0.43-1.45) | 0.421 | 0.83(0.49-1.42) | 0.495 |
| Fatigue | Not | - | - | - | - |
|  | Yes | 1.95(1.42-2.67) | <0.001 | 1.91(1.32-2.77) | <0.001 |
| RFP | Wild-type* | - | - | - | - |
|  | rpoB_p.Ser450 | 1.80(1.09-2.98) | 0.023 | 2.20(1.20-4.07) | 0.011 |

Note: Each variable is taken the first level as the reference group;

Wild-type* indicates that no mutation was detected.


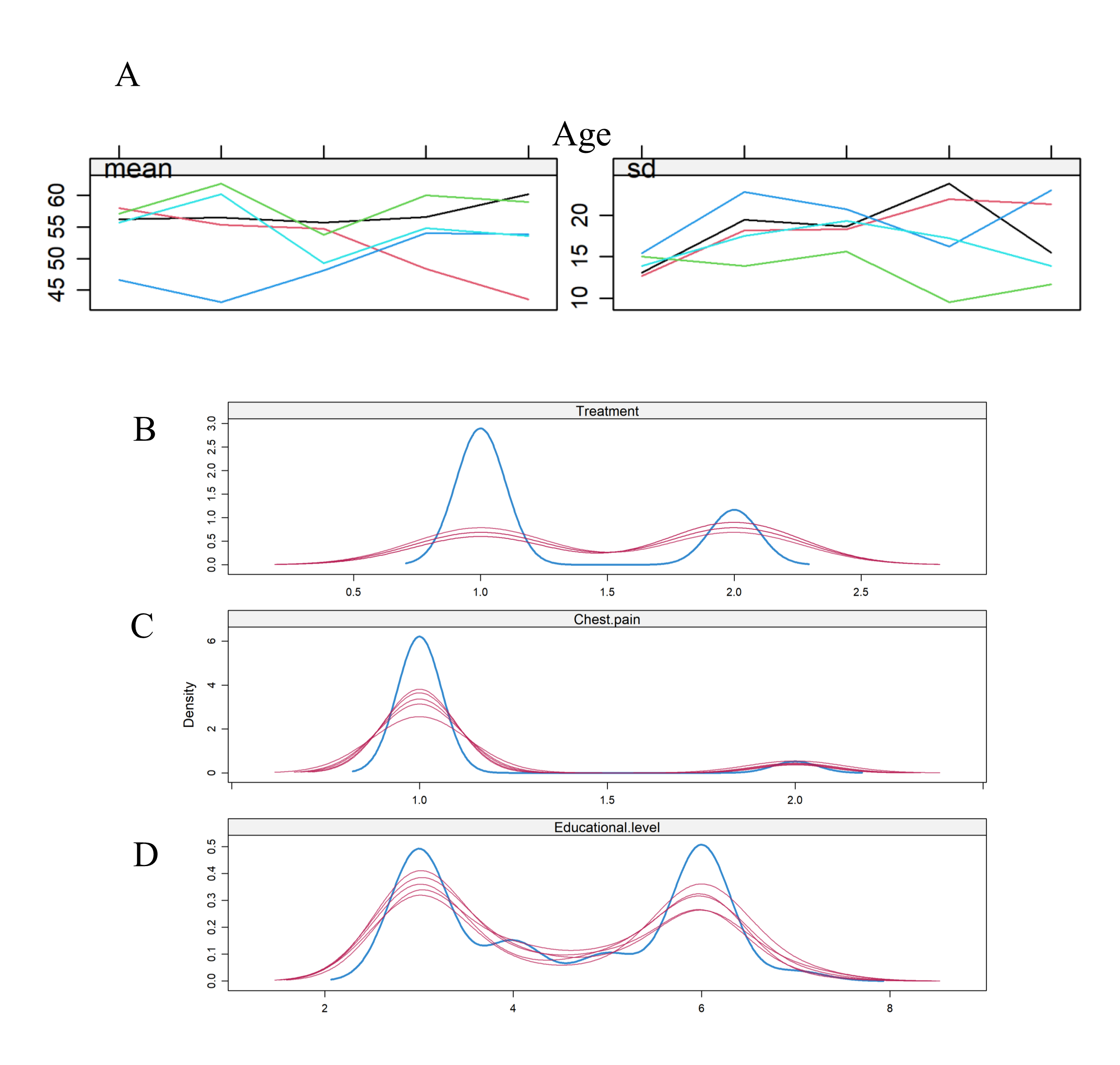


**Figure S1. Distribution of key variables after multiple imputation**

(A) Mean and standard deviation (SD) trends of age after multiple imputation.

(B) Distribution densities of treatment after multiple imputation.

(C) Distribution densities of Chest.pain after multiple imputation.

(D) Distribution densities of Educational level after multiple imputation.

**Table S3 (5 sub-tables in total)**

**Table S3.1 Mutation sites and the number of strains resistant to rifampicin**

| Mutation sites | Numbers |
| --- | --- |
| rpoB_p.Ser450Leu | 107 |
| rpoB_p.His445Asp | 18 |
| rpoB_p.His445Tyr | 15 |
| rpoB_p.Leu430Pro | 15 |
| rpoB_p.Leu452Pro | 14 |
| rpoB_p.Asp435Val | 6 |
| rpoB_p.His445Arg | 5 |
| rpoB_p.His445Asn | 4 |
| rpoB_p.Asp435Gly | 3 |
| rpoB_p.Ser450Phe | 1 |
| rpoB_p.Ser441Leu | 2 |
| rpoB_p.Asp435Tyr | 2 |
| rpoB_p.Ser450Asn | 1 |
| rpoB_p.Ser450Gln | 2 |
| rpoB_p.Ser441Ala | 1 |
| rpoB_p.Gln432Pro | 1 |
| rpoB_p.His445Leu | 1 |
| rpoB_p.His445Pro | 1 |
| rpoB_p.Ser450Trp | 2 |
| rpoB_p.His445Gln | 1 |
| rpoB_p.Thr427Ala | 1 |
| rpoB_p.His445Ser | 2 |
| rpoB_p.Val170Phe | 2 |
| rpoB_c.1291_1293dupAGC | 1 |
| rpoB_c.1329_1331dupGAC | 1 |
| rpoB_p.Leu464Met rpoB_p.Ser441Ala | 1 |
| rpoB_p.His445Asn  rpoB_p.Leu430Pro | 2 |
| rpoB_p.Arg448Gln rpoB_p.His445Arg | 1 |
| rpoB_p.Ile480Val rpoB_p.Ser450Leu | 1 |
| rpoB_p.Ser450Leu rpoB_p.Thr400Ala | 1 |
| rpoB_p.Asp435Gly rpoB_p.Leu452Pro | 1 |
| rpoB_p.Glu761Asp rpoB_p.Ser450Leu | 1 |
| rpoB_p.Ser450Leu rpoC_p.Leu527Val | 1 |
| rpoB_p.Asp435Glu  rpoB_p.Ser441Leu | 1 |
| rpoB_p.Ser450Leu rpoC_p.Gly332Arg | 1 |
| rpoB_p.Leu430Pro rpoB_p.Asp435Asn | 1 |
| rpoB_p.Ser450Leu rpoB_p.His445Asp | 1 |
| rpoB_p.His445Pro rpoB_p.Lys446Gln | 1 |
| rpoB_p.Thr400Ala rpoB_p.Ser450Leu | 1 |
| rpoB_p.Leu452Pro rpoB_p.Asp435Gly | 1 |
| rpoB_p.His445Asn rpoB_p.Phe424Leu | 1 |
| rpoB_p.His445Gln rpoB_p.Leu430Pro | 4 |
| rpoB_p.Leu430Pro rpoB_p.His445Gln | 2 |
| rpoB_p.Asp435Glu rpoB_p.His445Asn | 1 |
| rpoB_p.Asp435Tyr rpoB_p.His445Asn | 1 |
| rpoB_p.Ser450Leu rpoC_p.Ile491Thr | 1 |
| rpoB_p.Asp435Gly rpoB_p.Ile491Phe | 1 |
| rpoB_p.Ser450Leu rpoC_p.Phe452Ser | 1 |
| rpoB_p.Asp435Gly rpoB_p.Leu430Pro | 2 |
| rpoB_p.Gln436His rpoB_p.His445Gln rpoB_p.Leu430Pro | 1 |
| rpoB_p.Leu443Ser ,rpoB_p.Ser450Leu | 1 |
| rpoB_p.Gln432Lys rpoB_p.His445Leu | 1 |
| rpoB_p.His445Pro rpoB_p.His445Ser rpoB_p.His445Tyr rpoB_p.Ser431Gly | 1 |

**Table S3.2 Mutation sites and the number of strains resistant to isoniazid**

| Mutation sites | Nnumbers |
| --- | --- |
| katG_p.Ser315Thr | 190 |
| inhA_c.-777C>T | 14 |
| katG_p.Ser315Asn | 14 |
| fabG1_c.-15C>T | 8 |
| ahpC_c.-52C>T | 6 |
| katG_p.Ile335Thr | 5 |
| katG_p.Asp735Ala | 2 |
| fabG1_c.-8T>C | 1 |
| inhA_c.-154G>A | 1 |
| katG_c.-330_41del | 1 |
| katG_c.463delT | 2 |
| katG_p.Trp191Arg | 1 |
| inhA_c.-778A>G | 1 |
| katG_p.Ser302Arg | 1 |
| katG_c.590_887del | 1 |
| katG_p.Trp91Arg | 1 |
| katG_p.Gln36* | 1 |
| katG_p.Lys143Thr | 1 |
| katG_c.-7296_*171del | 1 |
| katG_c.-10A>C | 2 |
| katG_p.Gly309Ser | 1 |
| katG_p.Tyr155Cys | 1 |
| katG_p.Trp161* | 1 |
| katG_p.Ser315 （AGC-AGT） | 1 |
| katG_p.Asp142Ala | 1 |
| katG_p.Asn138Asp | 1 |
| ahpC_c.-48G>A | 1 |
| ahpC_c.-81C>T | 1 |
| katG_p.Gln127Pro | 1 |
| katG_p.Ser315Gly | 1 |
| kasA_p.Gly312Ser | 2 |
| fabG1_c.-8T>C katG_p.Ser315Thr | 1 |
| fabG1_c.-15C>T katG_p.Ser315Thr | 1 |
| fabG1_c.-15C>T inhA_p.Ile194Thr | 1 |
| ahpC_c.-48G>A katG_c.1079delG | 1 |
| inhA_c.-777C>T katG_p.Glu289Asp | 1 |
| inhA_c.-154G>A katG_p.Ser315Thr | 1 |
| katG_p.Ile317Leu katG_p.Ile335Thr | 4 |
| ahpC_c.-52C>T katG_p.Gln461Pro | 1 |
| inhA_c.-777C>T katG_p.Gln127Pro | 1 |
| ahpC_c.-48G>A katG_p.Ser315Gly | 1 |
| ahpC_c.-48G>A katG_p.Ser315Gly | 1 |
| inhA_c.-154G>A katG_p.Gln127Pro | 1 |
| katG_p.Ile317Leu katG_p.Ile335Thr | 1 |
| inhA_p.Ser94Ala fabG1_c.-15C>T | 2 |
| katG_p.Gln127Pro fabG1_c.-17G>T | 1 |
| inhA_p.Ile194Thr fabG1_c.-15C>T | 1 |
| ahpC_c.-81C>T ahpC_c.-54C>T | 1 |
| kasA_p.Gly312Ser fabG1_c.-15C>T | 1 |
| ahpC_c.-81C>T katG_c.1195_1199dupGAACA | 1 |
| ahpC_c.-52C>T katG_c.1602dupG katG_p.Ser315Asn | 1 |
| katG_p.Ser315Arg katG_p.Ser315Ile katG_p.Ala281Val | 1 |
| katG_c.297delG katG_p.Ala379Val katG_p.Ile335Thr katG_c.303_306delGTTTinsTCTTC | 1 |

**Table S3.3 Mutation sites and the number of strains resistant to pyrazinamide**

| Mutation sites | Numbers |
| --- | --- |
| pncA_p.Ala146Thr | 3 |
| pncA_p.Ile90Ser | 3 |
| pncA_c.-11A>G | 2 |
| pncA_c.390_391dupGG | 2 |
| pncA_p.Asp136Gly | 2 |
| pncA_c.327_*333del | 1 |
| pncA_p.Lys96Thr | 1 |
| pncA_p.Thr61Pro | 1 |
| pncA_p.His137Pro | 1 |
| pncA_p.Thr142Met | 1 |
| pncA_c.244_245insT | 1 |
| pncA_p.Asp8Asn | 1 |
| pncA_p.Cys14Tyr | 1 |
| pncA_p.Ala134Val | 1 |
| pncA_p.Met1? | 1 |
| pncA_p.Pro62Arg | 1 |
| pncA_p.Thr76Pro | 1 |
| pncA_p.Val7Ala | 1 |
| pncA_p.Val7Gly | 1 |
| pncA_p.Pro54Gln | 1 |
| pncA_c.-345_*2862del | 1 |
| pncA_c.391dupG | 2 |
| pncA_p.Gln141* | 1 |
| pncA_p.Gly97Arg | 1 |
| pncA_c.-2011_*380del | 1 |
| pncA_p.Asp129Asn | 1 |
| pncA_p.Ala3Glu | 1 |
| pncA_p.Gly108Arg | 1 |
| pncA_p.Ile5Ser | 1 |
| pncA_p.Val139Leu | 1 |
| pncA_p.Gln10Pro | 1 |
| pncA_p.Leu4Ser | 1 |
| pncA_p.Tyr34Ser | 1 |
| pncA_p.Met175Ile pncA_c.-11T>C | 1 |
| pncA_p.Cys14Gly pncA_p.Tyr64Asp | 1 |
| pncA_p.Pro54Leu pncA_p.Thr100Ile pncA_p.Trp119Cys | 1 |

**Table S3.4 Mutation sites and the number of strains resistant to ethambutol**

| Mutation sites | Numbers |
| --- | --- |
| embB_p.Met306Val | 31 |
| embB_p.Met306Ile | 18 |
| embB_p.Gly406Ala | 4 |
| embB_p.Gly406Asp | 2 |
| embB_p.Gln497Lys | 2 |
| embA_c.-16C>T | 2 |
| embA_c.-12C>T | 1 |
| embB_p.Met306Leu | 1 |
| embB_p.Tyr319Cys | 1 |
| embB_p.Gly406Cys | 1 |
| embB_p.Ala659Thr | 1 |
| embB_p.Gln497Arg | 1 |
| embB_p.Tyr334His | 1 |
| embB_p.Asp328Tyr | 1 |
| embB_p.Asp1024Asn | 1 |
| embB_p.Asp354Ala | 2 |
| embB_p.Tyr319Ser | 1 |
| embA_c.-16C>G | 1 |
| embB_p.His1002Arg | 1 |
| embC_p.Gly272Ser | 1 |
| embA_c.[？]C>A | 1 |
| embA_c.-12C>T | 1 |
| embB_p.His312Arg | 1 |
| embB_p.Leu370Arg | 1 |
| embA_c.-11C>A embB_p.Asp354Ala | 1 |
| embB_p.Asp1024Asn embB_p.Gln497Arg | 1 |
| embB_p.His312Arg embB_p.Leu359Ile | 4 |
| embB_p.Met306Ile embB_p.Met306Leu | 1 |
| embB_p.His312Arg embB_p.Leu359Ile | 1 |
| embA_c.-12C>T embB_p.Met306Val | 1 |
| embB_p.Ala659Thr  embB_p.Tyr384Asn | 1 |
| embB_p.Glu378Ala embC_p.Thr270Ile | 6 |
| embB_p.Met306Ile embA_c.-12C>T | 1 |
| embB_p.Met306Ile embB_p.Gly406Ala | 1 |
| embB_p.Asp328Gly embB_p.Met306Val | 1 |
| embB_p.Met306Val embB_p.Tyr319Cys | 1 |
| embB_p.Met306Val embB_p.Asp1024Asn | 1 |
| embB_p.Gln497Pro embA_c.-12C>T | 1 |
| embC_p.Thr270Ile embB_p.Glu378Ala | 1 |
| embB_p.Asp354Ala embB_p.Asp1024Asn | 1 |
| embB_p.Ala388Gly embB_p.Ala659Thr embB_p.Asn399Asp embB_p.His312Arg embB p.Leu359Ile | 2 |
| embB_p.His312Arg embB_p.Leu359Ile embB_p.Tyr334His | 1 |
| embB_p.His312Arg embB_p.Thr437Ala embB_p.Tyr384Asn | 1 |
| embB_p.Ala388Gly embB_p.Ala659Thr embB_p.Asn399Asp embB_p.His312Arg embB p.Leu359Ile embB_p.Tyr384Asn | 1 |
| embB_p.Ala388Gly embB_p.Ala659Thr embB_p.Asn399Asp embB_p.His312Arg embB_p.Leu359Ile embB_p.Tyr384Asn | 1 |
| embB_p.Ala388Gly embB_p.Ala659Thr embB_p.Asn399Asp embB_p.His312Arg embB p.Leu359Ile embB_p.Met306Val embB_p.Tyr384Asn | 1 |
| embB_p.Met306Ile embB_p.Gly406Cys embB_p.Gln497Pro | 1 |

**Table S3.5 Mutation sites and the number of strains resistant to streptomycin**

| Mutation sites | Numbers |
| --- | --- |
| rpsL_p.Lys43Arg | 59 |
| rrs_n.514A>C | 19 |
| rrs_n.462C>T | 15 |
| rpsL_p.Lys88Arg | 11 |
| rrs_n.517C>T | 6 |
| rrs_n.1401A>G | 3 |
| gid_c.351delG | 2 |
| gid_c.18_19insT | 1 |
| gid_c.357delC | 1 |
| gid_c.267_270dupTCTC | 1 |
| gid_c.115delC | 1 |
| gid_c.102delG | 1 |
| gid_p.Glu60* | 1 |
| gid_c.386delG | 1 |
| rrs_n.888G>A | 1 |
| gid_c.102delG rpsL_p.Lys43Arg | 1 |
| rpsL_p.Lys88Arg rrs_n.462C>T rrs_n.799C>T | 1 |
| rrs_n.190G>A rrs n.462C>T | 1 |
| rpsL_p.Lys43Arg rrs_n.1401A>G | 1 |
| rpsL_p.Lys43Arg rrs_n.514A>C | 1 |
| rpsL_p.Lys43Arg rrs_n.462C>T | 1 |

**Table S4 (6 sub-tables in total)**

**Table S4.1 Summary of drug-resistant strains of RFP + INH + PZA type**

| Strain number | RFP | INH | PZA |
| --- | --- | --- | --- |
| 203627-1 | rpoB_p.Ser450Leu | katG_p.Ser315Thr | pncA_c.327_*333del |
| 2303201336 | rpoB_p.Ser441Leu | fabG1_c.-8T>C katG_p.Ser315Thr | pncA_p.Lys96Thr |
| 2303221532 | rpoB_p.Ser450Leu | katG_p.Ser315Thr | pncA_p.Thr61Pro |
| 2303241615 | rpoB_p.Asp435Gly | fabG1_c.-15C>T katG_p.Ser315Thr | pncA_c.-11A>G |
| 238218 | rpoB_p.His445Asp | katG_p.Ser315Asn | pncA_p.His137Pro |
| 569 | rpoB_p.His445Gln rpoB_p.Leu430Pro | katG_p.Ser315Thr | pncA_c.244_245insT |
| GL240041 | rpoB_p.Asp435Val | katG_p.Ser315Thr | pncA_p.Ala134Val |
| GL240102 | rpoB_p.Ser450Leu | katG_p.Ser315Thr | pncA_p.Met1? |
| GL240312 | rpoB_p.His445Asp | ahpC_c.-52C>T | pncA_c.390_391dupGG |
| GL240577 | rpoB_p.Asp435Gly rpoB_p.Ile491Phe | katG_p.Ser315Thr | pncA_c.-11A>G |
| GL240583 | rpoB_p.Ser450Leu | katG_p.Ser315Thr | pncA_p.Ala146Thr |
| GL240611 | rpoB_p.Ser450Leu | katG_p.Ser315Thr | pncA_p.Ala146Thr |
| GL240866 | rpoB_p.Ser450Leu | katG_p.Ser315Thr | pncA_p.Thr76Pro |
| GL250031 | rpoB_p.Ser450Leu | katG_p.Ser315Thr | pncA_p.Val7Ala |
| GL250090 | rpoB_p.Ser450Leu | katG_p.Ser315Thr | pncA_p.Ala146Thr |
| GL250091 | rpoB_p.Ser450Leu | katG_p.Ser315Thr | pncA_p.Val7Gly |
| GL250217 | rpoB_p.Ser450Leu | katG_p.Ser315Asn | pncA_p.Pro54Leu pncA_p.Thr100Ile pncA_p.Trp119Cys |
| HZ20250035 | rpoB_p.Ser450Leu | katG_p.Ser315Thr | pncA_p.Pro54Gln |
| LB250143 | rpoB_p.Ser450Leu | katG_p.Ser315Thr | pncA_c.-345_*2862del |
| LB240673 | rpoB_p.Ser450Leu | katG_p.Ser315Thr | pncA_c.391dupG |
| LB240851 | rpoB_p.Ser450Leu | katG_p.Ser315Thr | pncA_c.390_391dupGG |
| FC20250039 | rpoB_p.Leu452Pro | katG_p.Ser315Thr | pncA_c.391dupG |
| NN20240049 | rpoB_p.His445Asn rpoB_p.Leu430Pro | katG_p.Ser315Thr | pncA_p.Gln141* |
| YL240504 | rpoB_p.Gln432Pro | katG_p.Ser315Thr | pncA_p.Gly97Arg |
| YL240874 | rpoB_p.Ser450Leu | katG_p.Ser315Thr | pncA_c.-2011_*380del |
| YL240915 | rpoB_p.Asp435Gly rpoB_p.Leu430Pro | katG_p.Ser315Thr | pncA_p.Ala3Glu |
| FC20250257 | rpoB_p.Ser450Leu rpoC_p.Gly332Arg | katG_p.Trp161* | pncA_p.Gly108Arg |
| GJ042 | rpoB_p.His445Pro | ahpC_c.-52C>T | pncA_p.Ile90Ser |
| GJ078 | rpoB_p.Ser450Leu | katG_p.Ser315Thr | pncA_p.Ile5Ser |
| GJ091 | rpoB_p.His445Tyr | katG_p.Ser315Thr | pncA_p.Cys14Gly pncA_p.Tyr64Asp |
| GJ110 | rpoB_p.Ser450Leu | katG_p.Ser315Thr | pncA_p.Val139Leu |
| GJ123 | rpoB_p.Thr400Ala rpoB_p.Ser450Leu | katG_p.Ser315Thr | pncA_p.Gln10Pro |
| GJ1407 | rpoB_p.Leu452Pro | katG_p.Ser315Thr | pncA_p.Tyr34Ser |

**Table S4.2 Summary of drug-resistant strains of RFP + INH + EMB type**

| Strain number | RFP | INH | EMB |
| --- | --- | --- | --- |
| 203627-1 | rpoB_p.Ser450Leu | katG_p.Ser315Thr | embA_c.-11C>A embB_p.Asp354Ala |
| 213355 | rpoB_p.Ser450Leu | katG_p.Ser315Thr | embB_p.Gly406Asp |
| 2303130940 | rpoB_p.Ser450Phe | katG_p.Ser315Thr | embB_p.Met306Ile |
| 2303151009 | rpoB_p.His445Arg | katG_p.Ser315Thr | embB_p.Met306Ile |
| 2303201336 | rpoB_p.Ser441Leu | fabG1_c.-8T>C katG_p.Ser315Thr | embB_p.Met306Val |
| 2303241615 | rpoB_p.Asp435Gly | fabG1_c.-15C>T katG_p.Ser315Thr | embB_p.Met306Leu |
| 238061 | rpoB_p.Ser450Leu | katG_p.Ser315Thr | embB_p.Met306Ile |
| 238218 | rpoB_p.His445Asp | katG_p.Ser315Asn | embB_p.Met306Ile |
| 239444 | rpoB_p.Ile480Val rpoB_p.Ser450Leu | katG_c.-330_41del | embB_p.Tyr319Cys |
| 239491 | rpoB_p.His445Gln rpoB_p.Leu430Pro | katG_p.Ser315Thr | embB_p.Gly406Cys |
| 240040CTAB | rpoB_p.Asp435Gly rpoB_p.Leu452Pro | katG_p.Ser315Thr | embB_p.Met306Val |
| CZ240887 | rpoB_p.Ser450Leu | katG_p.Ser315Thr | embB_p.Met306Val |
| 569 | rpoB_p.His445Gln rpoB_p.Leu430Pro | katG_p.Ser315Thr | embB_p.Asp1024Asn embB_p.Gln497Arg |
| FC20250237 | rpoB_p.Leu452Pro | katG_p.Ser315Asn | embB_p.Met306Val |
| FC20250304 | rpoB_p.Leu452Pro | katG_p.Ser315Asn | embB_p.Met306Val |
| GL240041 | rpoB_p.Asp435Val | katG_p.Ser315Thr | embB_p.Gly406Asp |
| GL240102 | rpoB_p.Ser450Leu | katG_p.Ser315Thr | embB_p.Gln497Arg |
| GL240280 | rpoB_p.Ser450Leu rpoC_p.Ile491Thr | katG_p.Ser315Thr | embB_p.Met306Ile embB_p.Met306Leu |
| GL240299 | rpoB_p.Ser450Trp | katG_p.Ser315Thr | embB_p.Met306Val |
| GL240309 | rpoB_p.Ser450Leu | katG_p.Ser315Thr | embB_p.Gln497Lys |
| GL240311 | rpoB_p.His445Asp | katG_p.Ser315Thr | embB_p.Met306Ile |
| GL240312 | rpoB_p.His445Asp | ahpC_c.-52C>T | embB_p.Met306Val |
| GL240577 | rpoB_p.Asp435Gly rpoB_p.Ile491Phe | katG_p.Ser315Thr | embB_p.Asp328Tyr |
| GL240583 | rpoB_p.Ser450Leu | katG_p.Ser315Thr | embB_p.Gly406Ala |
| GL240611 | rpoB_p.Ser450Leu | katG_p.Ser315Thr | embB_p.Gly406Ala |
| GL240786 | rpoB_p.Asp435Val | inhA_c.-777C>T katG_p.Gln127Pro | embB_p.Met306Ile |
| GL240866 | rpoB_p.Ser450Leu | katG_p.Ser315Thr | embB_p.Met306Ile |
| GL241018 | rpoB_p.His445Tyr | katG_p.Ser315Thr | embB_p.Asp1024Asn |
| GL250031 | rpoB_p.Ser450Leu | katG_p.Ser315Thr | embB_p.Met306Val |
| GL250060 | rpoB_p.Asp435Gly | katG_p.Ser315Thr | embB_p.Met306Val |
| GL250090 | rpoB_p.Ser450Leu | katG_p.Ser315Thr | embB_p.Gly406Ala |
| GL250094 | rpoB_p.Ser450Leu | inhA_c.-777C>T | embA_c.-12C>T embB_p.Met306Val |
| GL250399 | rpoB_p.His445Asp | katG_p.Ser315Thr | embB_p.Asp354Ala |
| HZ20250035 | rpoB_p.Ser450Leu | katG_p.Ser315Thr | embB_p.Met306Val |
| HZ20250037 | rpoB_p.Ser450Trp | katG_p.Ser315Thr | embB_p.Met306Val |
| HZ20250038 | rpoB_p.Ser450Leu | katG_p.Ser315Thr | embB_p.Met306Ile |
| HZ20250041 | rpoB_p.Ser450Leu | inhA_c.-777C>T | embB_p.Met306Val |
| LB241298 | rpoB_p.Asp435Gly rpoB_p.Leu430Pro | katG_p.Ser315Thr | embB_p.Met306Val |
| LB250143 | rpoB_p.Ser450Leu | katG_p.Ser315Thr | embB_p.Met306Val |
| LB240673 | rpoB_p.Ser450Leu | katG_p.Ser315Thr | embB_p.Met306Val |
| LB240851 | rpoB_p.Ser450Leu | katG_p.Ser315Thr | embB_p.Tyr319Ser |
| FC20250039 | rpoB_p.Leu452Pro | katG_p.Ser315Thr | embB_p.Met306Ile |
| NN20240049 | rpoB_p.His445Asn  rpoB_p.Leu430Pro | katG_p.Ser315Thr | embB_p.Met306Ile |
| YL240261 | rpoB_p.Ser450Leu | katG_p.Ser315Thr | embB_p.Met306Val |
| YL240286 | rpoB_p.His445Tyr | katG_p.Ser315Thr | embB_p.Met306Val |
| YL240293 | rpoB_p.Ser450Leu | katG_p.Ser315Thr | embB_p.Met306Val |
| YL240708 | rpoB_p.Ser450Leu | ahpC_c.-52C>T | embB_p.Met306Val |
| YL240732 | rpoB_p.Ser450Leu | katG_p.Ser315Thr | embB_p.Met306Val |
| YL240874 | rpoB_p.Ser450Leu | katG_p.Ser315Thr | embB_p.His1002Arg |
| YL240915 | rpoB_p.Asp435Gly rpoB_p.Leu430Pro | katG_p.Ser315Thr | embB_p.Met306Val |
| FC20250257 | rpoB_p.Ser450Leu rpoC_p.Gly332Arg | katG_p.Trp161* | embB_p.Met306Val |
| GJ024 | rpoB_p.Leu430Pro rpoB_p.Asp435Asn | katG_p.Ser315Thr | embB_p.Met306Val |
| GJ025 | rpoB_p.His445Pro rpoB_p.Lys446Gln | katG_p.Ser315Thr | embB_p.Met306Val |
| GJ035 | rpoB_p.His445Leu | katG_p.Ser315Thr | embA_c.[?]C>A |
| GJ039 | rpoB_p.Ser450Leu | katG_p.Ser315Asn | embB_p.Met306Ile |
| GJ078 | rpoB_p.Ser450Leu | katG_p.Ser315Thr | embB_p.Met306Val |
| GJ086 | rpoB_p.His445Tyr | katG_p.Ser315Thr | embA_c.-16C>T |
| GJ091 | rpoB_p.His445Tyr | katG_p.Ser315Thr | embB_p.Met306Ile embA_c.-12C>T |
| GJ095 | rpoB_p.Ser450Leu | katG_p.Ser315Thr | embB_p.Asp328Gly embB_p.Met306Val |
| GJ099 | rpoB_p.Asp435Val | katG_p.Ser315Asn | embB_p.Met306Val embB_p.Tyr319Cys |
| GJ123 | rpoB_p.Thr400Ala rpoB_p.Ser450Leu | katG_p.Ser315Thr | embB_p.Met306Val |
| GJ128 | rpoB_p.Asp435Val | katG_p.Ser315Thr | embB_p.Met306Val |
| GJ138 | rpoB_p.Ser450Leu | katG_p.Ser315Thr | embB_p.Met306Ile |
| GJ150 | rpoB_p.Ser450Leu | katG_p.Ser315Thr | embB_p.Met306Val |
| GJ154 | rpoB_p.Leu452Pro rpoB_p.Asp435Gly | katG_p.Ser315Thr | embB_p.Met306Ile |
| GJ158 | rpoB_p.Ser450Leu | katG_p.Ser315Thr | embB_p.Met306Val embB_p.Asp1024Asn |
| GJ175 | rpoB_p.His445Asp | katG_p.Ser315Asn | embB_p.Met306Ile |
| GJ341 | rpoB_p.Ser450Leu | ahpC_c.-52C>T | embB_p.Gln497Pro embA_c.-12C>T |
| GJ1407 | rpoB_p.Leu452Pro | katG_p.Ser315Thr | embB_p.Asp354Ala embB_p.Asp1024Asn |

**Table S4.3 Summary of drug-resistant strains of RFP + INH +S type**

| Strain number | RFP | INH | S |
| --- | --- | --- | --- |
| 203627-1 | rpoB_p.Ser450Leu | katG_p.Ser315Thr | rpsL_p.Lys88Arg |
| 2303221532 | rpoB_p.Ser450Leu | katG_p.Ser315Thr | rpsL_p.Lys43Arg |
| 2303241615 | rpoB_p.Asp435Gly | fabG1_c.-15C>T katG_p.Ser315Thr | rpsL_p.Lys88Arg |
| 2304140738 | rpoB_p.Arg448Gln rpoB_p.His445Arg | katG_p.Ser315Thr | rpsL_p.Lys43Arg |
| 239562 | rpoB_p.Ser450Leu rpoB_p.Thr400Ala | fabG1_c.-15C>T inhA_p.Ile194Thr | rrs_n.514A>C |
| 240040CTAB | rpoB_p.Asp435Gly rpoB_p.Leu452Pro | katG_p.Ser315Thr | rrs_n.517C>T |
| Y42 | rpoB_p.Ser450Leu | katG_p.Asp735Ala | rpsL_p.Lys43Arg |
| 569 | rpoB_p.His445Gln rpoB_p.Leu430Pro | katG_p.Ser315Thr | rpsL_p.Lys43Arg |
| CZ240883 | rpoB_p.His445Asp | katG_p.Ser315Thr | rpsL_p.Lys43Arg |
| GL240041 | rpoB_p.Asp435Val | katG_p.Ser315Thr | rpsL_p.Lys88Arg |
| GL240102 | rpoB_p.Ser450Leu | katG_p.Ser315Thr | rpsL_p.Lys43Arg |
| GL240299 | rpoB_p.Ser450Trp | katG_p.Ser315Thr | gid_c.18_19insT |
| GL240311 | rpoB_p.His445Asp | katG_p.Ser315Thr | rpsL_p.Lys43Arg |
| GL240464 | rpoB_p.Ser450Leu | katG_p.Ser315Thr | rpsL_p.Lys43Arg |
| GL240503 | rpoB_p.His445Asp | katG_p.Ser315Thr | rpsL_p.Lys88Arg |
| GL240577 | rpoB_p.Asp435Gly rpoB_p.Ile491Phe | katG_p.Ser315Thr | rrs_n.517C>T |
| GL240583 | rpoB_p.Ser450Leu | katG_p.Ser315Thr | rrs_n.514A>C |
| GL240586 | rpoB_p.Asp435Tyr | katG_p.Ser315Thr | rpsL_p.Lys43Arg |
| GL240611 | rpoB_p.Ser450Leu | katG_p.Ser315Thr | rrs_n.514A>C |
| GL240866 | rpoB_p.Ser450Leu | katG_p.Ser315Thr | rpsL_p.Lys43Arg |
| GL241018 | rpoB_p.His445Tyr | katG_p.Ser315Thr | rrs_n.514A>C |
| GL250031 | rpoB_p.Ser450Leu | katG_p.Ser315Thr | rpsL_p.Lys43Arg |
| GL250090 | rpoB_p.Ser450Leu | katG_p.Ser315Thr | rrs_n.514A>C |
| GL250091 | rpoB_p.Ser450Leu | katG_p.Ser315Thr | rpsL_p.Lys43Arg |
| GL250094 | rpoB_p.Ser450Leu | inhA_c.-777C>T | rpsL_p.Lys43Arg |
| GL250394 | rpoB_p.Ser450Leu | katG_p.Ser315Thr | rpsL_p.Lys43Arg |
| GL250399 | rpoB_p.His445Asp | katG_p.Ser315Thr | rpsL_p.Lys43Arg |
| HZ20250041 | rpoB_p.Ser450Leu | inhA_c.-777C>T | rpsL_p.Lys43Arg |
| LB241095 | rpoB_p.His445Ser | katG_p.Ser315Thr | rpsL_p.Lys43Arg |
| LB250143 | rpoB_p.Ser450Leu | katG_p.Ser315Thr | rrs_n.517C>T |
| LB250157 | rpoB_p.Leu452Pro | katG_p.Ser315Thr | rrs_n.514A>C |
| LB240673 | rpoB_p.Ser450Leu | katG_p.Ser315Thr | gid_c.102delG rpsL_p.Lys43Arg |
| LB240851 | rpoB_p.Ser450Leu | katG_p.Ser315Thr | gid_c.115delC |
| FC20250039 | rpoB_p.Leu452Pro | katG_p.Ser315Thr | rpsL_p.Lys43Arg |
| NN20240049 | rpoB_p.His445Asn rpoB_p.Leu430Pro | katG_p.Ser315Thr | rrs_n.514A>C |
| YL240194 | rpoB_p.Ser450Leu | katG_p.Ser315Thr | rpsL_p.Lys43Arg |
| YL240261 | rpoB_p.Ser450Leu | katG_p.Ser315Thr | rpsL_p.Lys88Arg rrs_n.462C>T rrs_n.799C>T |
| YL240286 | rpoB_p.His445Tyr | katG_p.Ser315Thr | rpsL_p.Lys43Arg |
| YL240293 | rpoB_p.Ser450Leu | katG_p.Ser315Thr | gid_c.102delG |
| YL240429 | rpoB_p.His445Tyr | katG_p.Ser315Thr | rpsL_p.Lys43Arg |
| YL240504 | rpoB_p.Gln432Pro | katG_p.Ser315Thr | rpsL_p.Lys88Arg |
| YL240710 | rpoB_p.His445Tyr | katG_p.Ser315Thr | rpsL_p.Lys43Arg |
| YL240732 | rpoB_p.Ser450Leu | katG_p.Ser315Thr | gid_p.Glu60* |
| YL240874 | rpoB_p.Ser450Leu | katG_p.Ser315Thr | rpsL_p.Lys43Arg |
| YL240900 | rpoB_p.His445Tyr | katG_p.Ser315Thr | rpsL_p.Lys43Arg |
| NN-F2024245 | rpoB_p.His445Ser | katG_p.Ser315Thr | rpsL_p.Lys43Arg |
| YL240915 | rpoB_p.Asp435Gly rpoB_p.Leu430Pro | katG_p.Ser315Thr | rrs_n.514A>C |
| FC20250246 | rpoB_p.Ser450Leu | katG_p.Ser315Thr | rpsL_p.Lys43Arg |
| GJ024 | rpoB_p.Leu430Pro rpoB_p.Asp435Asn | katG_p.Ser315Thr | rpsL_p.Lys43Arg |
| GJ011 | rpoB_p.Leu452Pro | katG_p.Ser315Thr | rpsL_p.Lys43Arg |
| GJ012 | rpoB_p.Ser450Leu | katG_p.Ser315Thr | rpsL_p.Lys43Arg |
| GJ025 | rpoB_p.His445Pro rpoB_p.Lys446Gln | katG_p.Ser315Thr | rpsL_p.Lys88Arg |
| GJ078 | rpoB_p.Ser450Leu | katG_p.Ser315Thr | rpsL_p.Lys43Arg |
| GJ091 | rpoB_p.His445Tyr | katG_p.Ser315Thr | rpsL_p.Lys43Arg |
| GJ095 | rpoB_p.Ser450Leu | katG_p.Ser315Thr | rpsL_p.Lys43Arg |
| GJ109 | rpoB_p.Ser450Leu | katG_p.Ser315Thr | rpsL_p.Lys88Arg |
| GJ110 | rpoB_p.Ser450Leu | katG_p.Ser315Thr | rpsL_p.Lys88Arg |
| GJ123 | rpoB_p.Thr400Ala rpoB_p.Ser450Leu | katG_p.Ser315Thr | rpsL_p.Lys43Arg rrs_n.514A>C |
| GJ154 | rpoB_p.Leu452Pro rpoB_p.Asp435Gly | katG_p.Ser315Thr | rrs_n.517C>T |
| GJ175 | rpoB_p.His445Asp | katG_p.Ser315Asn | rpsL_p.Lys43Arg |
| GJ200 | rpoB_p.Ser450Leu | katG_p.Ser315Thr | rpsL_p.Lys43Arg |

**Table S4.4 Summary of drug-resistant strains of RFP + INH + PZA+EMB type**

| Strain number | RFP | INH | PZA | EMB |
| --- | --- | --- | --- | --- |
| 203627-1 | rpoB_p.Ser450Leu | katG_p.Ser315Thr | pncA_c.327_*333del | embA_c.-11C>A embB_p.Asp354Ala |
| 2303201336 | rpoB_p.Ser441Leu | fabG1_c.-8T>C katG_p.Ser315Thr | pncA_p.Lys96Thr | embB_p.Met306Val |
| 2303241615 | rpoB_p.Asp435Gly | fabG1_c.-15C>T katG_p.Ser315Thr | pncA_c.-11A>G | embB_p.Met306Leu |
| 238218 | rpoB_p.His445Asp | katG_p.Ser315Asn | pncA_p.His137Pro | embB_p.Met306Ile |
| 569 | rpoB_p.His445Gln rpoB_p.Leu430Pro | katG_p.Ser315Thr | pncA_c.244_245insT | embB_p.Asp1024Asn embB_p.Gln497Arg |
| GL240041 | rpoB_p.Asp435Val | katG_p.Ser315Thr | pncA_p.Ala134Val | embB_p.Gly406Asp |
| GL240102 | rpoB_p.Ser450Leu | katG_p.Ser315Thr | pncA_p.Met1? | embB_p.Gln497Arg |
| GL240312 | rpoB_p.His445Asp | ahpC_c.-52C>T | pncA_c.390_391dupGG | embB_p.Met306Val |
| GL240577 | rpoB_p.Asp435Gly rpoB_p.Ile491Phe | katG_p.Ser315Thr | pncA_c.-11A>G | embB_p.Asp328Tyr |
| GL240583 | rpoB_p.Ser450Leu | katG_p.Ser315Thr | pncA_p.Ala146Thr | embB_p.Gly406Ala |
| GL240611 | rpoB_p.Ser450Leu | katG_p.Ser315Thr | pncA_p.Ala146Thr | embB_p.Gly406Ala |
| GL240866 | rpoB_p.Ser450Leu | katG_p.Ser315Thr | pncA_p.Thr76Pro | embB_p.Met306Ile |
| GL250031 | rpoB_p.Ser450Leu | katG_p.Ser315Thr | pncA_p.Val7Ala | embB_p.Met306Val |
| GL250090 | rpoB_p.Ser450Leu | katG_p.Ser315Thr | pncA_p.Ala146Thr | embB_p.Gly406Ala |
| HZ20250035 | rpoB_p.Ser450Leu | katG_p.Ser315Thr | pncA_p.Pro54Gln | embB_p.Met306Val |
| LB250143 | rpoB_p.Ser450Leu | katG_p.Ser315Thr | pncA_c.-345_*2862del | embB_p.Met306Val |
| LB240673 | rpoB_p.Ser450Leu | katG_p.Ser315Thr | pncA_c.391dupG | embB_p.Met306Val |
| LB240851 | rpoB_p.Ser450Leu | katG_p.Ser315Thr | pncA_c.390_391dupGG | embB_p.Tyr319Ser |
| FC20250039 | rpoB_p.Leu452Pro | katG_p.Ser315Thr | pncA_c.391dupG | embB_p.Met306Ile |
| NN20240049 | rpoB_p.His445Asn  rpoB_p.Leu430Pro | katG_p.Ser315Thr | pncA_p.Gln141* | embB_p.Met306Ile |
| YL240874 | rpoB_p.Ser450Leu | katG_p.Ser315Thr | pncA_c.-2011_*380del | embB_p.His1002Arg |
| YL240915 | rpoB_p.Asp435Gly rpoB_p.Leu430Pro | katG_p.Ser315Thr | pncA_p.Ala3Glu | embB_p.Met306Val |
| FC20250257 | rpoB_p.Ser450Leu rpoC_p.Gly332Arg | katG_p.Trp161* | pncA_p.Gly108Arg | embB_p.Met306Val |
| GJ078 | rpoB_p.Ser450Leu | katG_p.Ser315Thr | pncA_p.Ile5Ser | embB_p.Met306Val |
| GJ091 | rpoB_p.His445Tyr | katG_p.Ser315Thr | pncA_p.Cys14Gly pncA_p.Tyr64Asp | embB_p.Met306Ile embA_c.-12C>T |
| GJ123 | rpoB_p.Thr400Ala rpoB_p.Ser450Leu | katG_p.Ser315Thr | pncA_p.Gln10Pro | embB_p.Met306Val |
| GJ1407 | rpoB_p.Leu452Pro | katG_p.Ser315Thr | pncA_p.Tyr34Ser | embB_p.Asp354Ala embB_p.Asp1024Asn |

**Table S4.5 Summary of drug-resistant strains of RFP + INH + PZA+ S type**

| Strain number | RFP | INH | PZA | S |
| --- | --- | --- | --- | --- |
| 203627-1 | rpoB_p.Ser450Leu | katG_p.Ser315Thr | pncA_c.327_*333del | rpsL_p.Lys88Arg |
| 2303221532 | rpoB_p.Ser450Leu | katG_p.Ser315Thr | pncA_p.Thr61Pro | rpsL_p.Lys43Arg |
| 2303241615 | rpoB_p.Asp435Gly | fabG1_c.-15C>T katG_p.Ser315Thr | pncA_c.-11A>G | rpsL_p.Lys88Arg |
| 569 | rpoB_p.His445Gln rpoB_p.Leu430Pro | katG_p.Ser315Thr | pncA_c.244_245insT | rpsL_p.Lys43Arg |
| GL240041 | rpoB_p.Asp435Val | katG_p.Ser315Thr | pncA_p.Ala134Val | rpsL_p.Lys88Arg |
| GL240102 | rpoB_p.Ser450Leu | katG_p.Ser315Thr | pncA_p.Met1? | rpsL_p.Lys43Arg |
| GL240577 | rpoB_p.Asp435Gly rpoB_p.Ile491Phe | katG_p.Ser315Thr | pncA_c.-11A>G | rrs_n.517C>T |
| GL240583 | rpoB_p.Ser450Leu | katG_p.Ser315Thr | pncA_p.Ala146Thr | rrs_n.514A>C |
| GL240611 | rpoB_p.Ser450Leu | katG_p.Ser315Thr | pncA_p.Ala146Thr | rrs_n.514A>C |
| GL240866 | rpoB_p.Ser450Leu | katG_p.Ser315Thr | pncA_p.Thr76Pro | rpsL_p.Lys43Arg |
| GL250031 | rpoB_p.Ser450Leu | katG_p.Ser315Thr | pncA_p.Val7Ala | rpsL_p.Lys43Arg |
| GL250090 | rpoB_p.Ser450Leu | katG_p.Ser315Thr | pncA_p.Ala146Thr | rrs_n.514A>C |
| GL250091 | rpoB_p.Ser450Leu | katG_p.Ser315Thr | pncA_p.Val7Gly | rpsL_p.Lys43Arg |
| LB250143 | rpoB_p.Ser450Leu | katG_p.Ser315Thr | pncA_c.-345_*2862del | rrs_n.517C>T |
| LB240673 | rpoB_p.Ser450Leu | katG_p.Ser315Thr | pncA_c.391dupG | gid_c.102delG  rpsL_p.Lys43Arg |
| LB240851 | rpoB_p.Ser450Leu | katG_p.Ser315Thr | pncA_c.390_391dupGG | gid_c.115delC |
| FC20250039 | rpoB_p.Leu452Pro | katG_p.Ser315Thr | pncA_c.391dupG | rpsL_p.Lys43Arg |
| NN20240049 | rpoB_p.His445Asn  rpoB_p.Leu430Pro | katG_p.Ser315Thr | pncA_p.Gln141* | rrs_n.514A>C |
| YL240504 | rpoB_p.Gln432Pro | katG_p.Ser315Thr | pncA_p.Gly97Arg | rpsL_p.Lys88Arg |
| YL240874 | rpoB_p.Ser450Leu | katG_p.Ser315Thr | pncA_c.-2011_*380del | rpsL_p.Lys43Arg |
| YL240915 | rpoB_p.Asp435Gly rpoB_p.Leu430Pro | katG_p.Ser315Thr | pncA_p.Ala3Glu | rrs_n.514A>C |
| GJ078 | rpoB_p.Ser450Leu | katG_p.Ser315Thr | pncA_p.Ile5Ser | rpsL_p.Lys43Arg |
| GJ091 | rpoB_p.His445Tyr | katG_p.Ser315Thr | pncA_p.Cys14Gly pncA_p.Tyr64Asp | rpsL_p.Lys43Arg |
| GJ110 | rpoB_p.Ser450Leu | katG_p.Ser315Thr | pncA_p.Val139Leu | rpsL_p.Lys88Arg |
| GJ123 | rpoB_p.Thr400Ala rpoB_p.Ser450Leu | katG_p.Ser315Thr | pncA_p.Gln10Pro | rpsL_p.Lys43Arg rrs_n.514A>C |

**Table S4.6 Summary of drug-resistant strains of RFP + INH + PZA + EMB + S type**

| Strain number | RFP | INH | PZA | EMB | S |
| --- | --- | --- | --- | --- | --- |
| 203627-1 | rpoB_p.Ser450Leu | katG_p.Ser315Thr | pncA_c.327_*333del | embA_c.-11C>A embB_p.Asp354Ala | rpsL_p.Lys88Arg |
| 2303241615 | rpoB_p.Asp435Gly | fabG1_c.-15C>T katG_p.Ser315Thr | pncA_c.-11A>G | embB_p.Met306Leu | rpsL_p.Lys88Arg |
| 569 | rpoB_p.His445Gln rpoB_p.Leu430Pro | katG_p.Ser315Thr | pncA_c.244_245insT | embB_p.Asp1024Asn embB_p.Gln497Arg | rpsL_p.Lys43Arg |
| GL240041 | rpoB_p.Asp435Val | katG_p.Ser315Thr | pncA_p.Ala134Val | embB_p.Gly406Asp | rpsL_p.Lys88Arg |
| GL240102 | rpoB_p.Ser450Leu | katG_p.Ser315Thr | pncA_p.Met1? | embB_p.Gln497Arg | rpsL_p.Lys43Arg |
| GL240577 | rpoB_p.Asp435Gly rpoB_p.Ile491Phe | katG_p.Ser315Thr | pncA_c.-11A>G | embB_p.Asp328Tyr | rrs_n.517C>T |
| GL240583 | rpoB_p.Ser450Leu | katG_p.Ser315Thr | pncA_p.Ala146Thr | embB_p.Gly406Ala | rrs_n.514A>C |
| GL240611 | rpoB_p.Ser450Leu | katG_p.Ser315Thr | pncA_p.Ala146Thr | embB_p.Gly406Ala | rrs_n.514A>C |
| GL240866 | rpoB_p.Ser450Leu | katG_p.Ser315Thr | pncA_p.Thr76Pro | embB_p.Met306Ile | rpsL_p.Lys43Arg |
| GL250031 | rpoB_p.Ser450Leu | katG_p.Ser315Thr | pncA_p.Val7Ala | embB_p.Met306Val | rpsL_p.Lys43Arg |
| GL250090 | rpoB_p.Ser450Leu | katG_p.Ser315Thr | pncA_p.Ala146Thr | embB_p.Gly406Ala | rrs_n.514A>C |
| LB250143 | rpoB_p.Ser450Leu | katG_p.Ser315Thr | pncA_c.-345_*2862del | embB_p.Met306Val | rrs_n.517C>T |
| LB240673 | rpoB_p.Ser450Leu | katG_p.Ser315Thr | pncA_c.391dupG | embB_p.Met306Val | gid_c.102delG rpsL_p.Lys43Arg |
| LB240851 | rpoB_p.Ser450Leu | katG_p.Ser315Thr | pncA_c.390_391dupGG | embB_p.Tyr319Ser | gid_c.115delC |
| FC20250039 | rpoB_p.Leu452Pro | katG_p.Ser315Thr | pncA_c.391dupG | embB_p.Met306Ile | rpsL_p.Lys43Arg |
| NN20240049 | rpoB_p.His445Asn   rpoB_p.Leu430Pro | katG_p.Ser315Thr | pncA_p.Gln141* | embB_p.Met306Ile | rrs_n.514A>C |
| YL240874 | rpoB_p.Ser450Leu | katG_p.Ser315Thr | pncA_c.-2011_*380del | embB_p.His1002Arg | rpsL_p.Lys43Arg |
| YL240915 | rpoB_p.Asp435Gly rpoB_p.Leu430Pro | katG_p.Ser315Thr | pncA_p.Ala3Glu | embB_p.Met306Val | rrs_n.514A>C |
| GJ078 | rpoB_p.Ser450Leu | katG_p.Ser315Thr | pncA_p.Ile5Ser | embB_p.Met306Val | rpsL_p.Lys43Arg |
| GJ091 | rpoB_p.His445Tyr | katG_p.Ser315Thr | pncA_p.Cys14Gly pncA_p.Tyr64Asp | embB_p.Met306Ile，embA_c.-12C>T | rpsL_p.Lys43Arg |
| GJ123 | rpoB_p.Thr400Ala rpoB_p.Ser450Leu | katG_p.Ser315Thr | pncA_p.Gln10Pro | embB_p.Met306Val | rpsL_p.Lys43Arg rrs_n.514A>C |

**Table S5. The sensitivity analysis results of the multivariate Logistic regression model under different cavity grouping definitions**

| **Variable** | **Level** | **Model 1** | ***P*** | **Model2** | ***P*** |
| --- | --- | --- | --- | --- | --- |
| Gender | Male | - | - | - | - |
|  | Female | 0.77 (0.56–1.06) | 0.108 | 0.72 (0.51–1.01) | 0.060 |
| Fever | Not | - | - | - | - |
|  | Yes | 1.28 (0.92–1.81) | 0.152 | 1.56 (1.13–2.16) | 0.007 |
| Fatigue | Not | - | - | - | - |
|  | Yes | 1.54 (1.10–2.17) | 0.013 | 1.96 (1.43–2.69) | <0.001 |
| RFP | Wild-type* | - | - | - | - |
|  | rpoB_p.Ser450 | 2.19 (1.27–3.92) | 0.006 | 1.78 (1.08–2.95) | 0.025 |
| Age | - | 1.01 (1.00–1.02) | 0.169 | 1.01 (1.00–1.02) | 0.007 |
| History of previous  tuberculosis treatment | Not | - | - | - | - |
|  | Yes | 1.07 (0.49–2.29) | 0.866 | 1.80 (0.85–3.77) | 0.12 |

Note: Each variable is taken the first level as the reference group;

Model 1 compares single cavities (1 cavity) with multiple cavities (≥ 2 cavities); Model 2 compares mild to moderate cavities (1–2 cavities) with severe cavities（≥3 cavities).

**
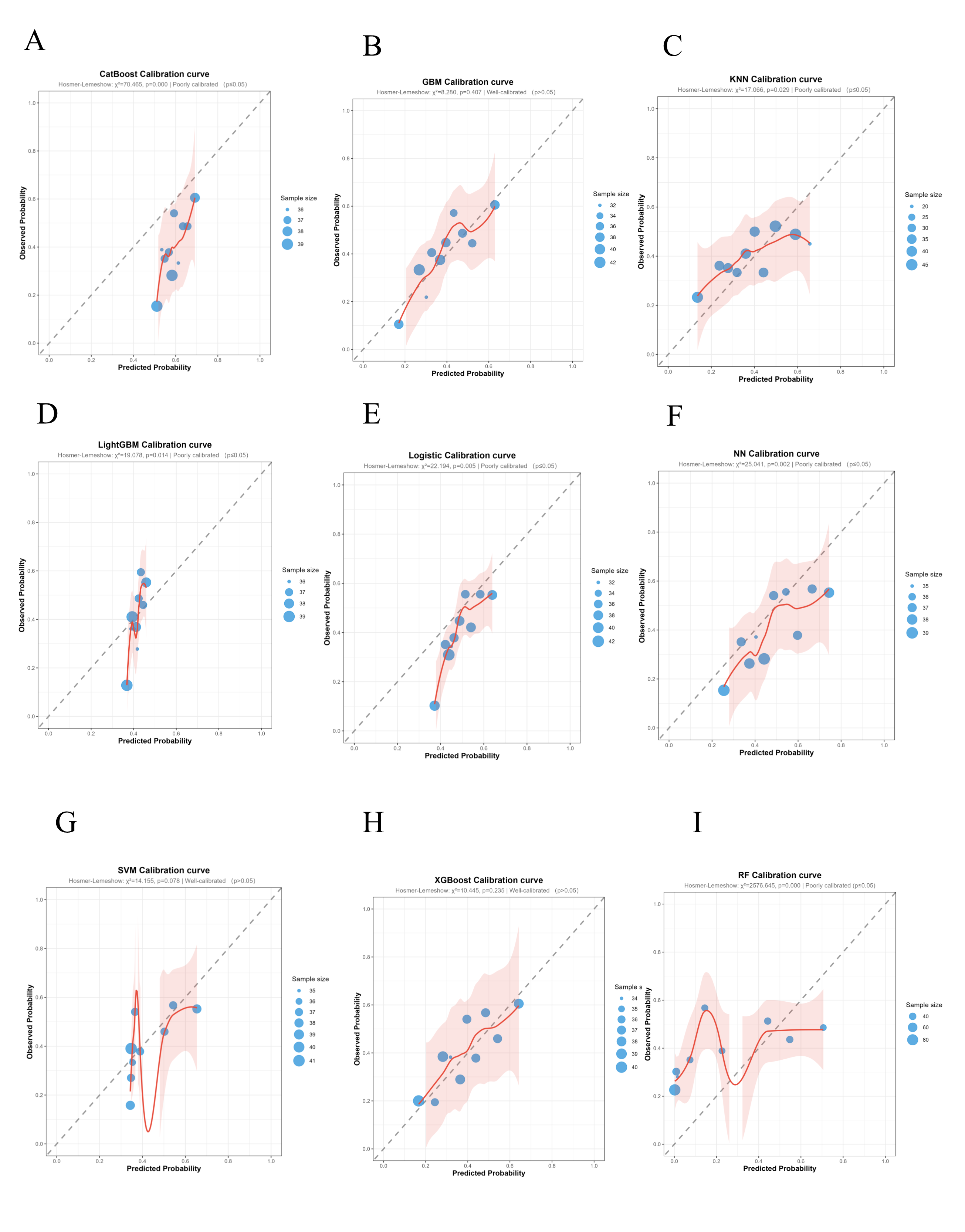
Figure S2. Nine machine learning model calibration curves**


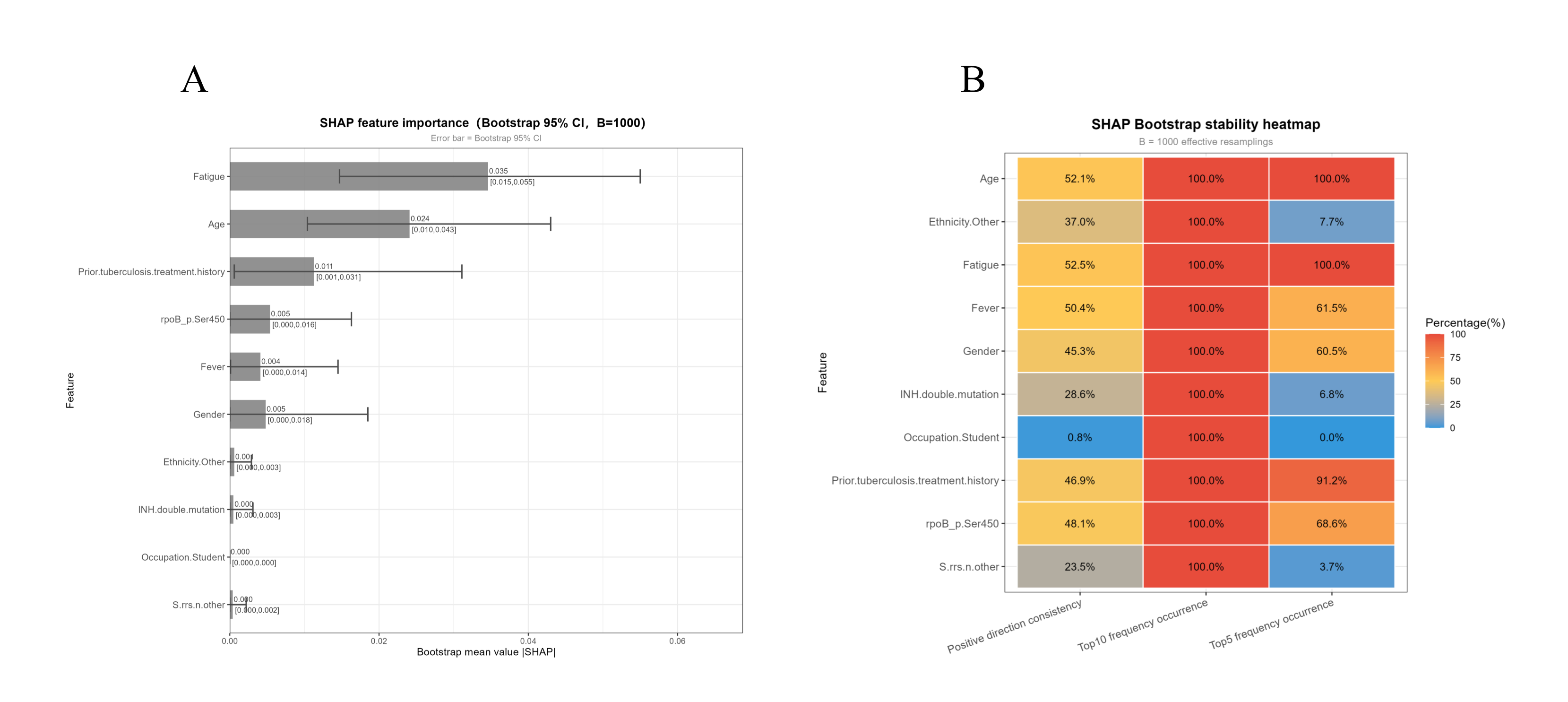


**Figure S3. SHAP-Based Feature Importance and Bootstrap Stability Analysis**

(A) SHAP Feature Importance (Bootstrap 95% CI)

(B) SHAP Bootstrap Stability Heatmap


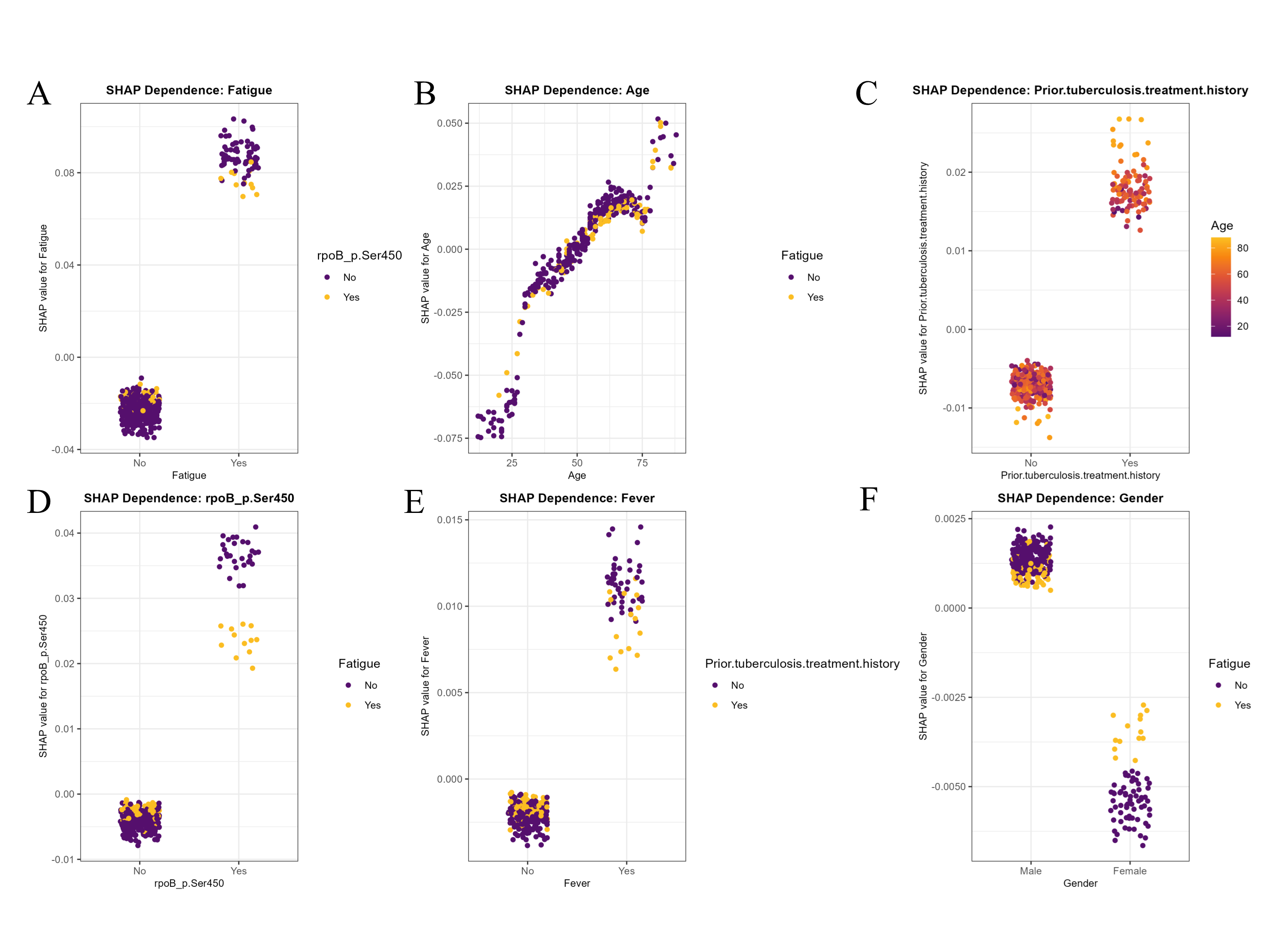


**Figure S4. SHAP Dependence Plots for Key Predictive Features**

(A) Dual feature dependence plot of fatigue with rpoB_p.Ser450.

(B) Dual feature dependence plot of age with fatigue.

(C) Dual feature dependence plot of prior tuberculosis treatment history with age.

(D) Dual feature dependence plot of rpoB_p.Ser450 with fatigue.

(E) Dual feature dependence plot of fever with prior tuberculosis treatment history.

(F) Dual feature dependence plot of gender with fatigue.
